# Supplementary material for: Assessment of STAT5 as a potential therapy target in enzalutamide-resistant prostate cancer
Source: PLoS One. 2020 Aug 13;15(8):e0237248. doi: 10.1371/journal.pone.0237248 (PMC7425943; doi:10.1371/journal.pone.0237248)

S1 Figure: Graphical description of the enzalutamide resistant cell models and analysis of STAT5 in enzalutamide-sensitive and -resistant xenograft models

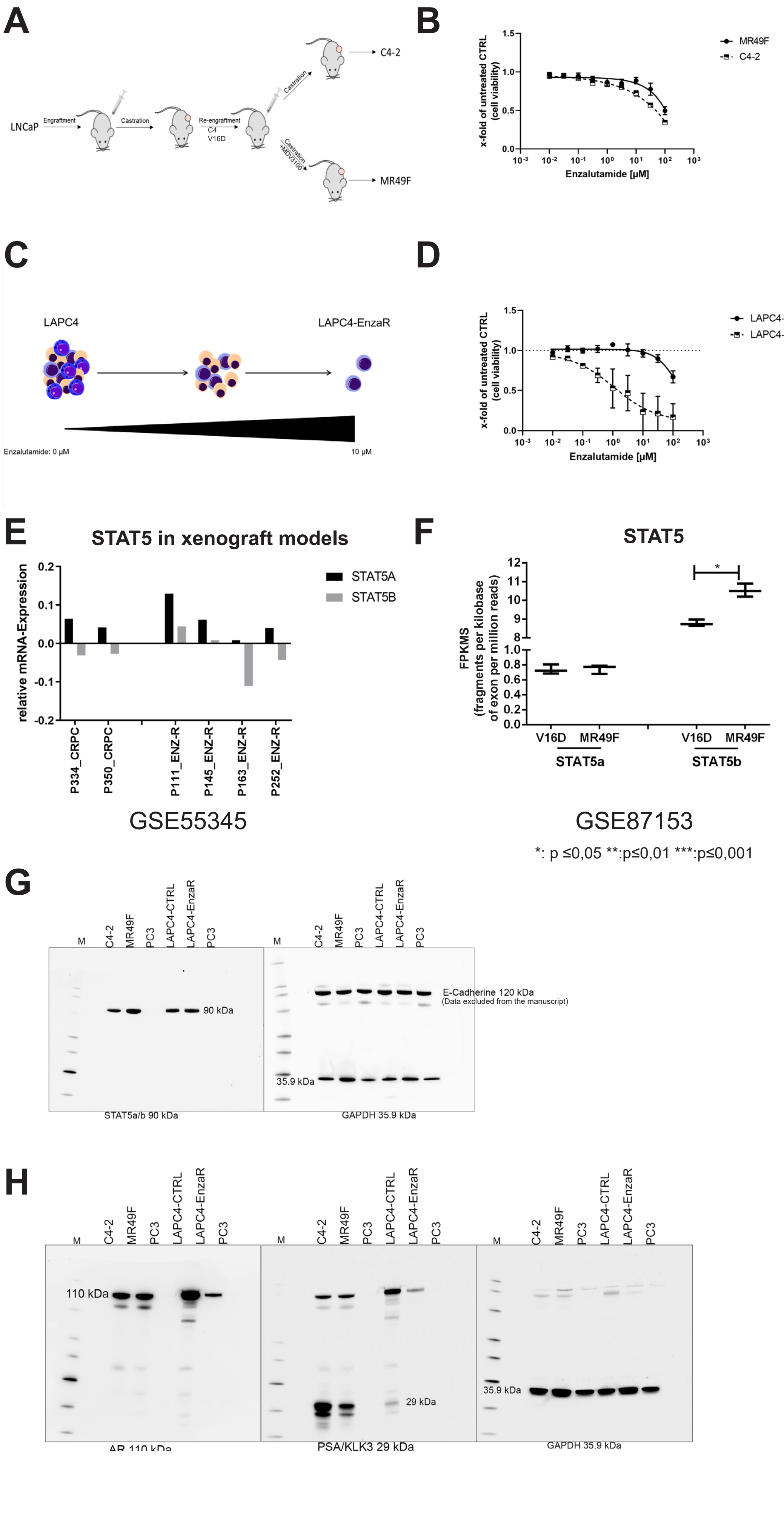

Supplement: S1 Fig — (A) Graphical description of the establishing process of C4-2 and MR49F cells. (B) Results of enzalutamide dose-response experiments in C4-2 and MR49F cells on cell viability 72 h after treatment. Cell viability was assessed by MTT assays. Data is shown as mean±s.e.m. of three independent experiments. (C) Graphical description of the establishing process of LAPC4-CTRL and LAPC4-EnzaR cells. (D) Results of enzalutamide dose-response experiments in LAPC4-CTRL and LAPC4-EnzaR cells on cell viability 72 h after treatment. Cell viability was assessed by MTT assays. Data is shown as mean±s.e.m. of three independent experiments. (E+F) STAT5a and STAT5b expression analysis of the public data set of the LNCaP-derived xenografts (E, GSE55345) and of the CRPC cell models V16D and MR49F (F, GSE87153) [7, 15, 40]. (G) Uncropped western blot images depicting STAT5 and GAPDH. (H) Uncropped western blot images depicting AR, PSA, and GAPDH. (PDF) [file pone.0237248.s001.pdf]
