# Supplementary figures and images for: Assessment of STAT5 as a potential therapy target in enzalutamide-resistant prostate cancer
Source: PLoS One. 2020 Aug 13;15(8):e0237248. doi: 10.1371/journal.pone.0237248 (PMC7425943; doi:10.1371/journal.pone.0237248)

**A**

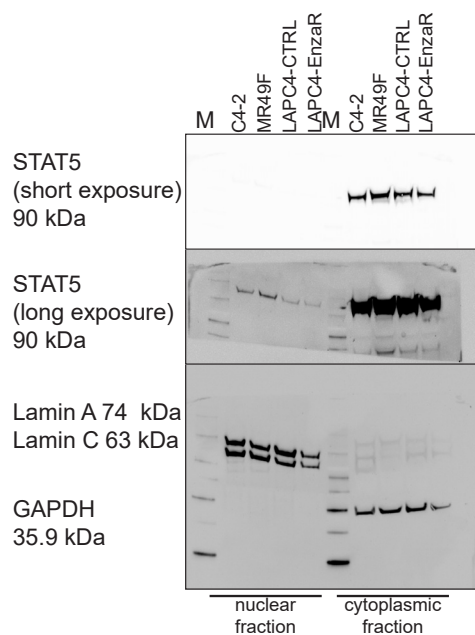

**B**

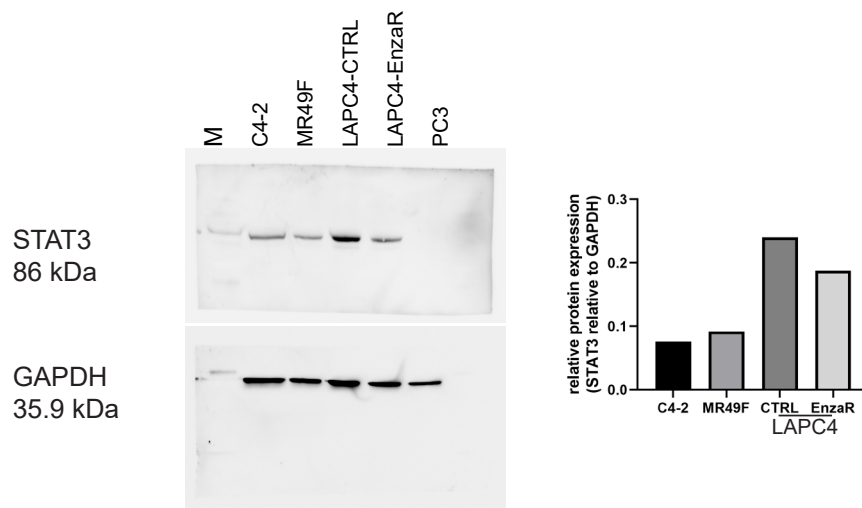

**C**

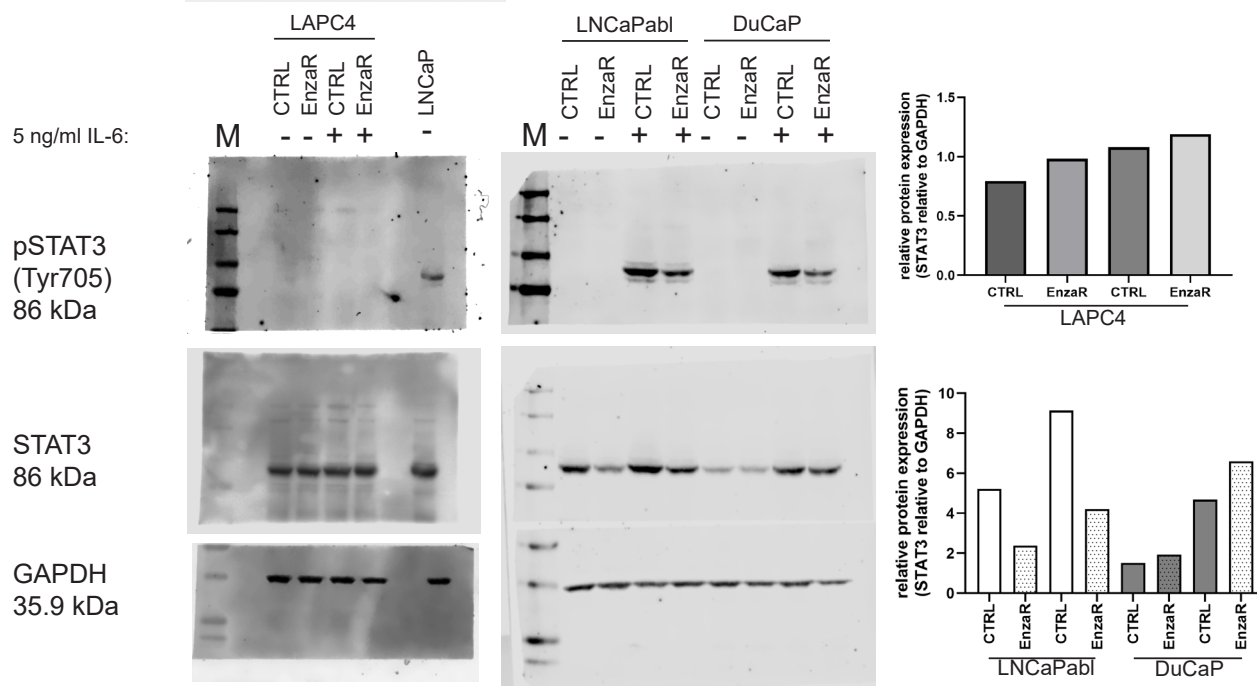

Supplement: S2 Fig — (A) Uncropped western blot of STAT5, Lamin A/C, and GAPDH after cytoplasmic and nuclear fractionation. Abbreviation: M: MagicMark XP. (B+C) Uncropped western blot images depicting pSTAT3, STAT3, GAPDH and densitometric analysis of STAT3 relative to GAPDH. (PDF) [file pone.0237248.s002.pdf]

S3 Figure: Western Blots STAT5 Activity in LNCaP Derived Models after Pimozide Treatment

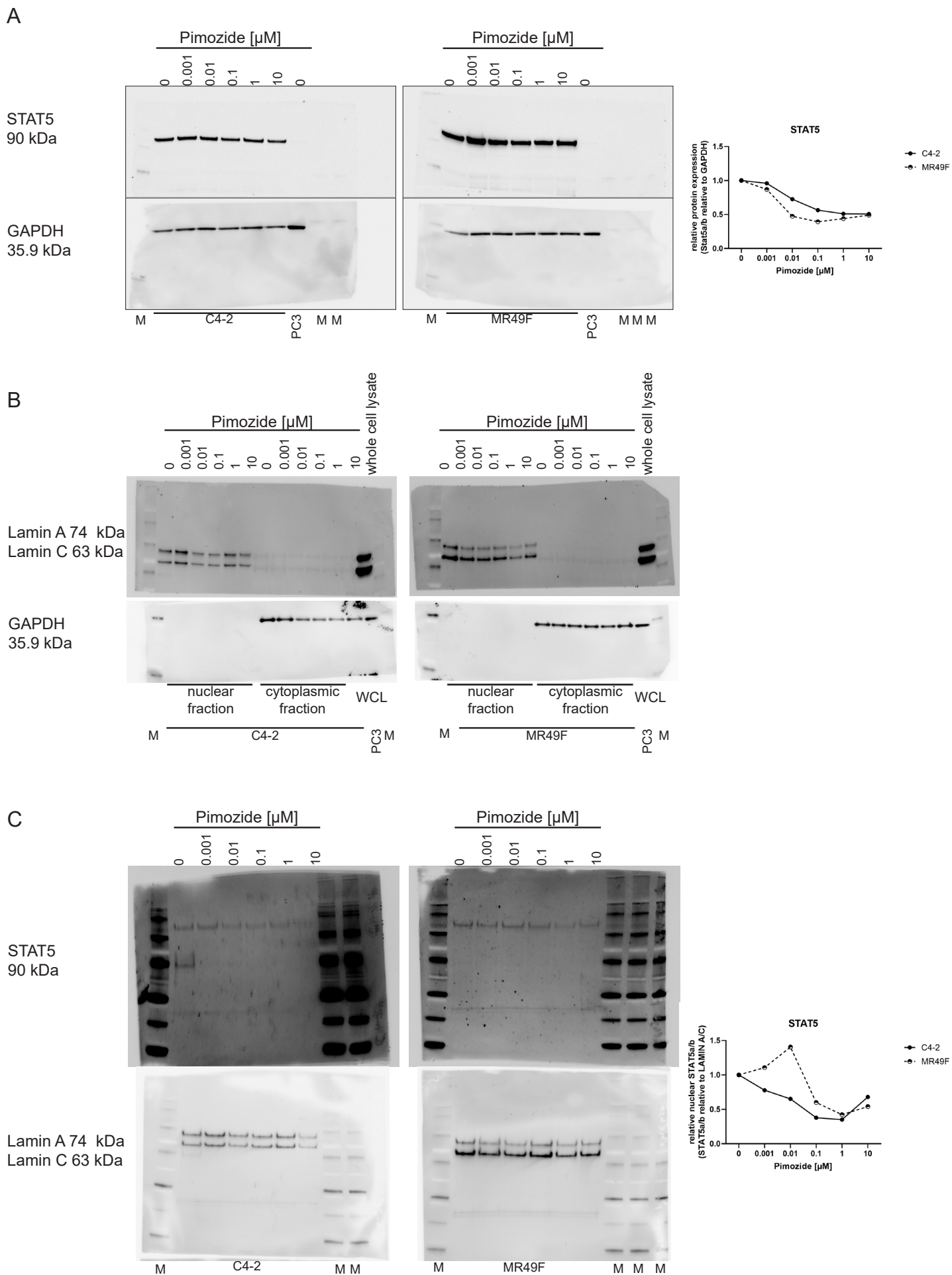

Supplement: S3 Fig — (A) Uncropped western blot images depicting STAT5 and GAPDH and densitometric analysis of STAT5 relative to GAPDH. (B) Uncropped western blot of Lamin A/C, and GAPDH after cytoplasmic and nuclear fractionation. (C) Uncropped western blot of Lamin A/C, and STAT5 after cytoplasmic and nuclear fractionation and densitometric analysis of nuclear STAT5 relative to Lamin A/C. Abbreviations: M: MagicMark XP; WCL: whole cell lysate. (PDF) [file pone.0237248.s003.pdf]

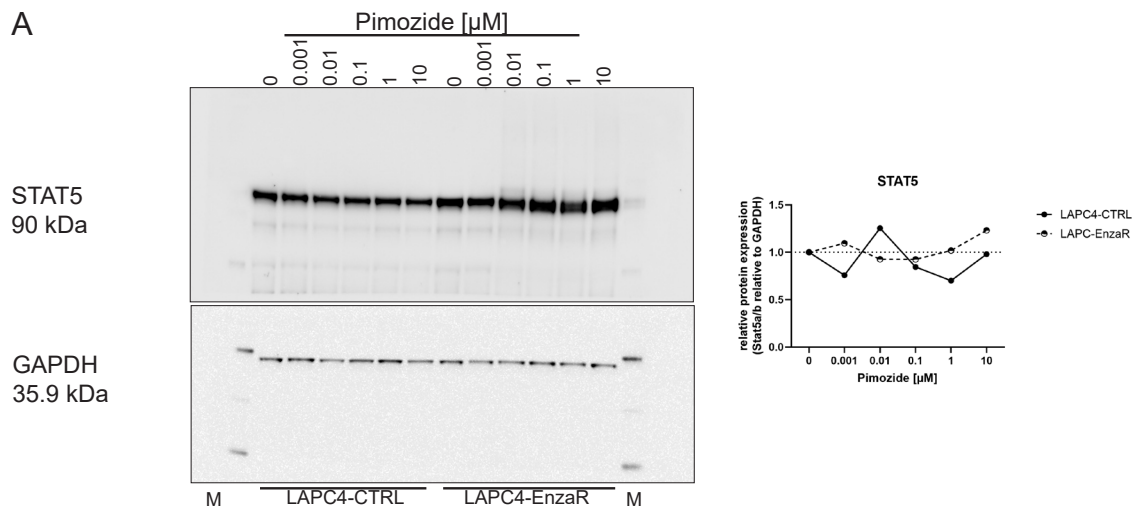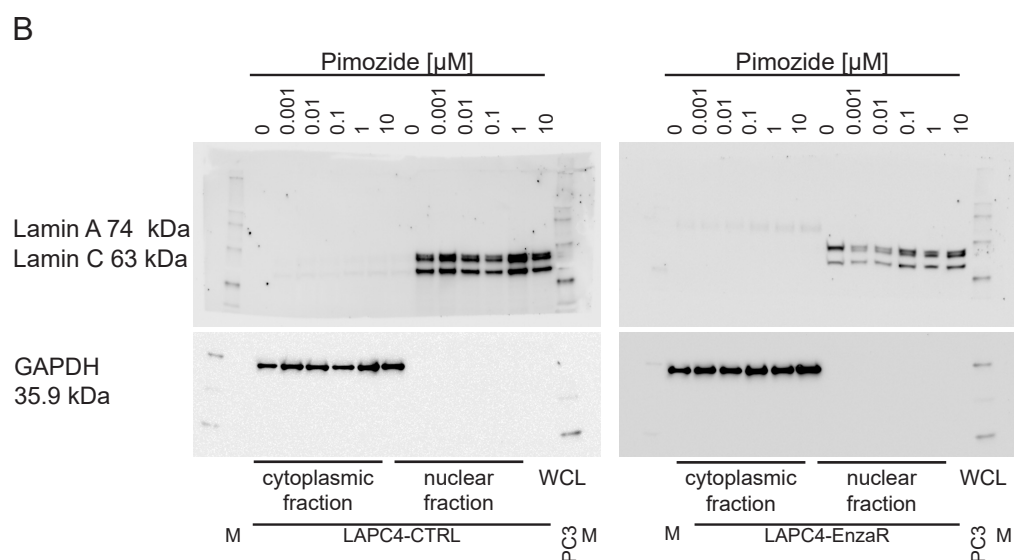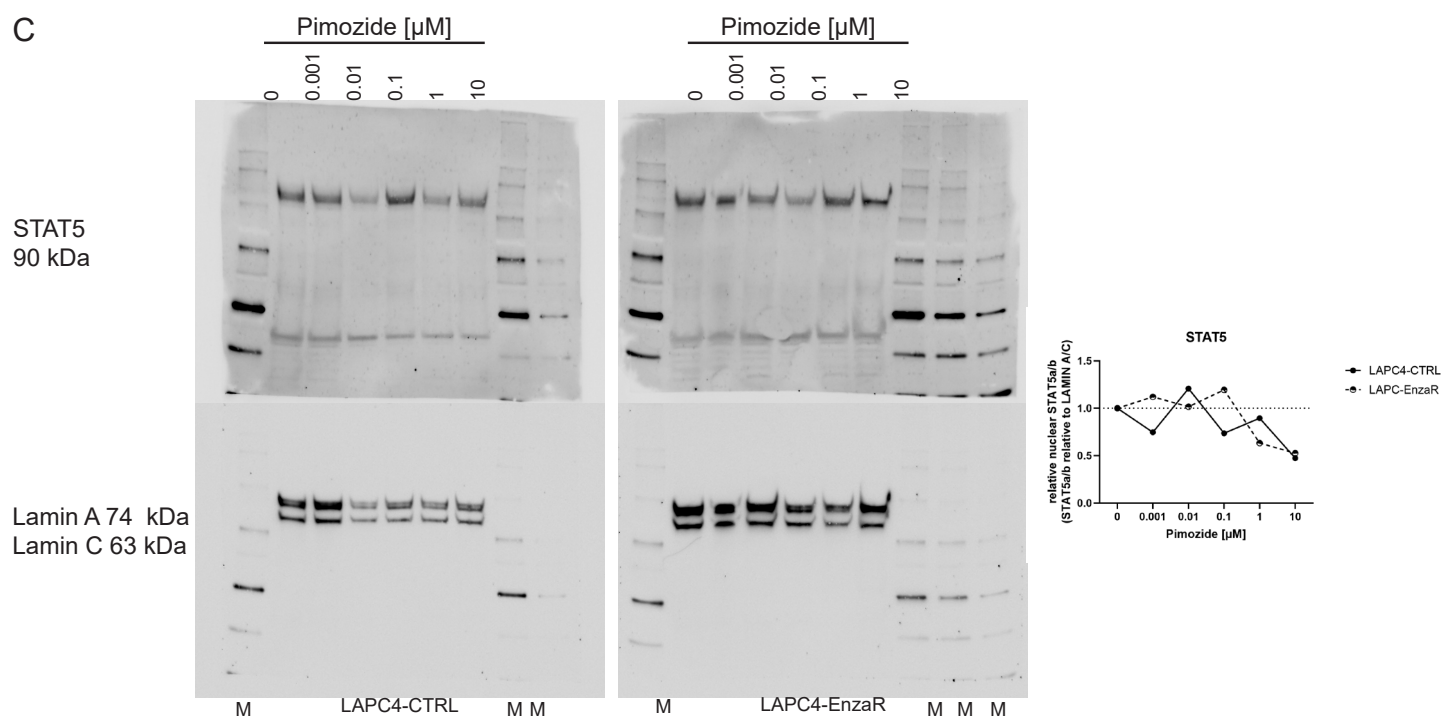

Supplement: S4 Fig — (A) Uncropped western blot images depicting STAT5 and GAPDH and densitometric analysis of STAT5 relative to GAPDH. (B) Uncropped western blot of Lamin A/C, and GAPDH after cytoplasmic and nuclear fractionation. (C) Uncropped western blot of Lamin A/C, and STAT5 after cytoplasmic and nuclear fractionation and densitometric analysis of nuclear STAT5 relative to Lamin A/C. Abbreviation: M: MagicMark XP. (PDF) [file pone.0237248.s004.pdf]

S5 Figure: Analysis of the relative AR and STAT5 activity after treatment

**A**

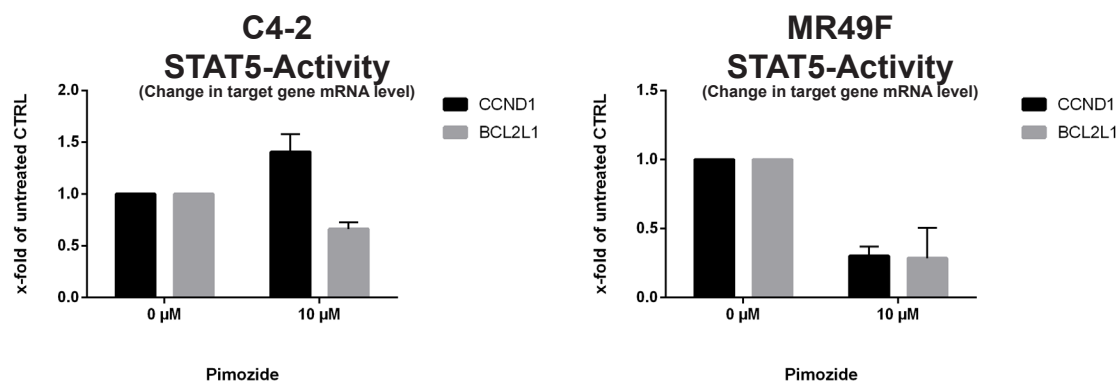

**B**

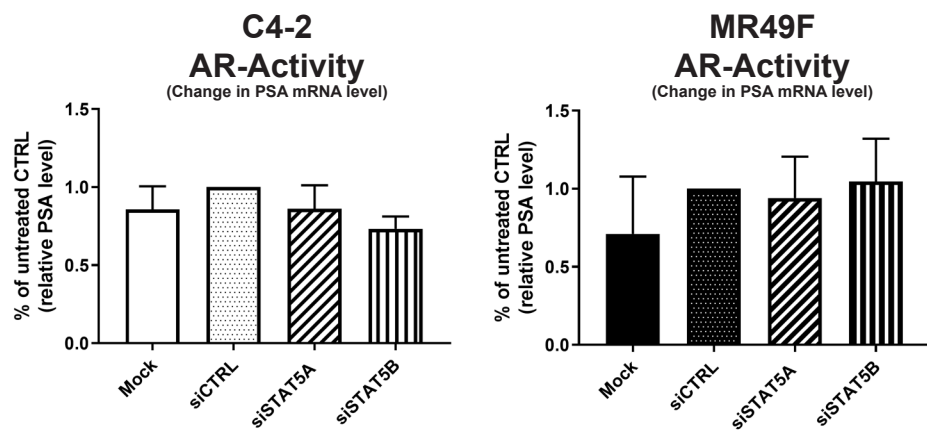

Supplement: S5 Fig — (A) qPCR analysis of Cyclin D1 (CCND1) and BCL-xL (BCL2L1) in C4-2 and MR49F cells treated with 10 μM Pimozide for 8 h. (B) qPCR analysis of PSA/KLK3 in C4-2 cells and MR49F cells transfected with siCTRL, siSTAT5a, and siSTAT5b for 24 h. (PDF) [file pone.0237248.s005.pdf]

06 Figure: Validation of STAT5a/b knock-down

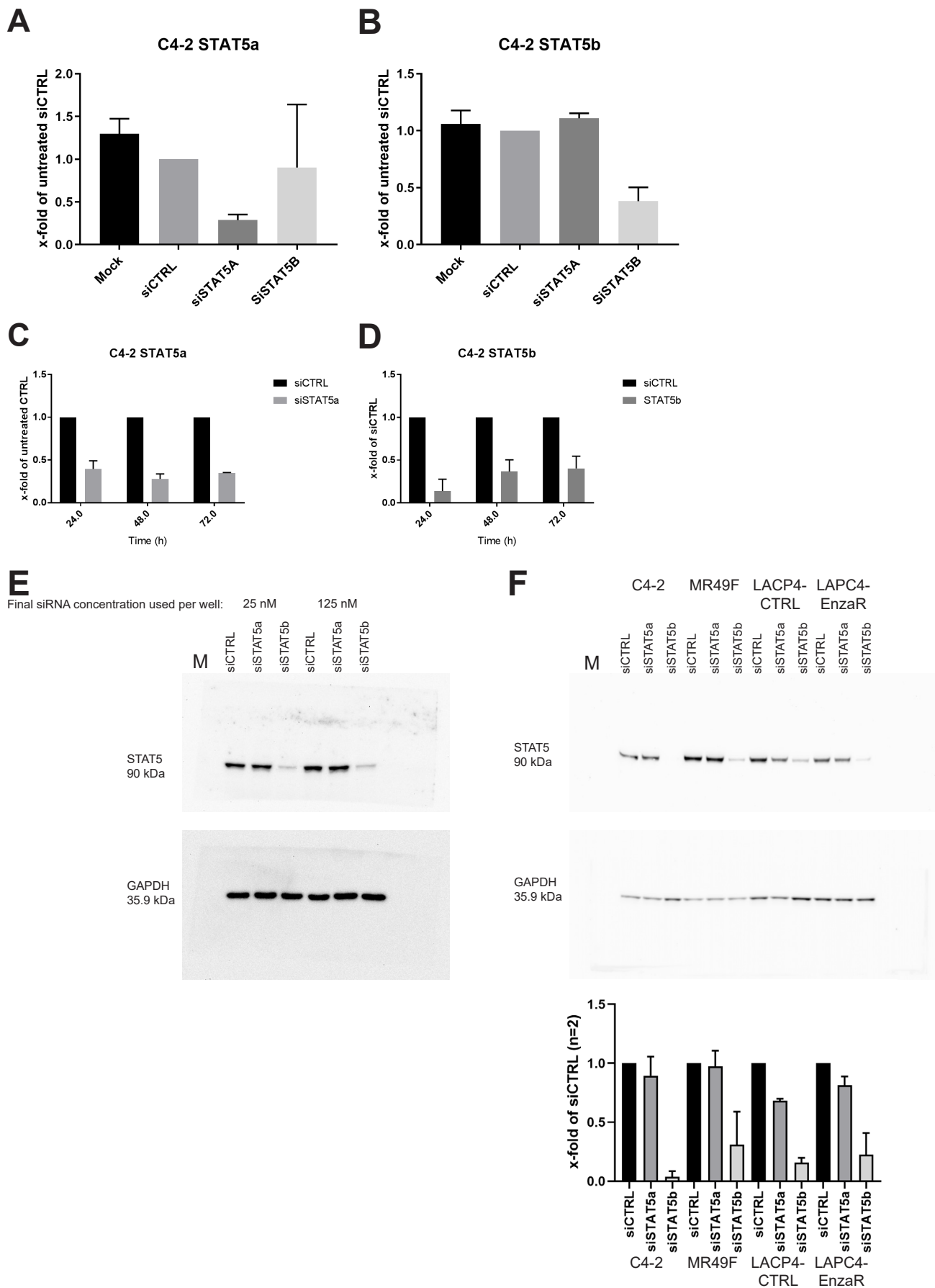

Supplement: S6 Fig — (A+B) qPCR analysis of STAT5a (A) and STAT5b (B) normalised to HPRT1 in C4-2 cells transfected with siCTRL, siSTAT5a, and siSTAT5b (all: final concentration 25 nM) for 48 h. (C+D) qPCR analysis of STAT5a (C) and STAT5b (D) in C4-2 cells transfected with siCTRL, siSTAT5a, and siSTAT5b (all: final concentration 25 nM) for 24 h, 48 h, and 72 h. (E) Western Blot of STAT5a/b and GAPDH in C4-2 cells after transfection with siCTRL, siSTAT5a, and siSTAT5b (all: final concentration 25 nM) for 48 h. (F) Western Blot of STAT5a/b and GAPDH in C4-2, MR49F, LAPC4-CTRL, LAPC4-EnzaR cells after transfection with siCTRL, siSTAT5a, and siSTAT5b (all: final concentration 25 nM) for 48 h. Abbreviation: M: MagicMark XP. (PDF) [file pone.0237248.s006.pdf]
