# Supplementary material for: Assessment of STAT5 as a potential therapy target in enzalutamide-resistant prostate cancer
Source: PLoS One. 2020 Aug 13;15(8):e0237248. doi: 10.1371/journal.pone.0237248 (PMC7425943; doi:10.1371/journal.pone.0237248)
Supplement: S1 Table — (DOCX) [file pone.0237248.s007.docx]

**S1 Table: Cell culture media for used cell lines**

| **Cell line** | **Medium** | | **Supplements** | | **Date of last authentication** | **Date of last mycoplasm test** |
| --- | --- | --- | --- | --- | --- | --- |
|  | **Name** | **Manufacturer** | **Name** | **Manufacturer** |  |  |
| C4-2 | DMEM (low Glucose) | Gibco/Invitrogen | 10% FCS | Gibco/Invitrogen | 26.02.2020 | 12.02.2020 |
|  |  |  | 20 mM HEPES | Gibco/Invitrogen |  |  |
|  |  |  | 1% GlutaMAX | Gibco/Invitrogen |  |  |
|  |  |  | 1% Pen/Strep | Gibco/Invitrogen |  |  |
| MR49F | RPMI | Gibco/Invitrogen | 10% FCS | Gibco/Invitrogen | 26.02.2020 | 12.02.2020 |
|  |  |  | 1% GlutaMAX | Gibco/Invitrogen |  |  |
|  |  |  | 1% Pen/Strep | Gibco/Invitrogen |  |  |
|  |  |  | 10 µM Enzalutamide | Astellas Pharma |  |  |
| LAPC4-CTRL | IMDM | Sigma Aldrich | 10% FCS | Gibco/Invitrogen | 13.03.2020 | 12.02.2020 |
|  |  |  | 1% GlutaMAX | Gibco/Invitrogen |  |  |
| LAPC4-EnzaR | IMDM | Sigma Aldrich | 10% FCS | Gibco/Invitrogen | 13.03.2020 | 12.02.2020 |
|  |  |  | 1% GlutaMAX | Gibco/Invitrogen |  |  |
|  |  |  | 10 µM Enzalutamide | Astellas Pharma |  |  |
| LNCaPabl-CTRL | RPMI | Gibco/Invitrogen | 10% charcoal stripped FCS | HyClone, GE Healthcare | 21.10.2019 | 17.10.2019 |
|  |  |  | 1% GlutaMAX | Gibco/Invitrogen |  |  |
| LNCaPabl-EnzaR | RPMI | Gibco/Invitrogen | 10% charcoal stripped FCS | HyClone, GE Healthcare | 21.10.2019 | 17.10.2019 |
|  |  |  | 1% GlutaMAX | Gibco/Invitrogen |  |  |
|  |  |  | 10 µM Enzalutamide | Astellas Pharma |  |  |
| DuCaP-CTRL | RPMI | Gibco/Invitrogen | 10% FCS | Gibco/Invitrogen | 21.10.2019 | 17.10.2019 |
|  |  |  | 1% GlutaMAX | Gibco/Invitrogen |  |  |
| DuCaP-EnzaR | RPMI | Gibco/Invitrogen | 10% FCS | Gibco/Invitrogen | 21.10.2019 | 17.10.2019 |
|  |  |  | 1% GlutaMAX | Gibco/Invitrogen |  |  |
|  |  |  | 10 µM Enzalutamide | Astellas Pharma |  |  |
