# Supplementary material for: Assessment of STAT5 as a potential therapy target in enzalutamide-resistant prostate cancer
Source: PLoS One. 2020 Aug 13;15(8):e0237248. doi: 10.1371/journal.pone.0237248 (PMC7425943; doi:10.1371/journal.pone.0237248)
Supplement: S2 Table — (DOCX) [file pone.0237248.s008.docx]

**S2 Table: Antibodies and used Dilutions**

| **Name** | **Company** | **Product Number** | **Working** |
| --- | --- | --- | --- |
|  |  |  | **Dilution** |
|  |  |  |  |
| STAT5 (C-17) | Santa Cruz Biotechnology | sc-835 | 1:500 |
| Androgen Receptor (D6F11) XP | Cell Signaling Technology | 5153S | 1:5000 |
| GAPDH (6C5) | EMD Millipore | CB1001 | 1:50000 |
| Lamin A/C (4C11) | Cell Signaling Technology | 4777T | 1:5000 |
| Phospho-Stat3 (Tyr705) (D3A7) XP | Cell Signaling Technology | 9145S | 1:1000 |
| Polyclonal Goat Anti-Rabbit IgG/HRP | Dako | P0217 | 1:1000 |
| Polyclonal Rabbit Anti-Mouse IgG/HRP | Dako | P0260 | 1:1000 |
| PSA/KLK3 (D6B1) | Cell Signaling Technology | 5365T | 1:2000 |
| STAT3 (124H6) | Cell Signaling Technology | 9139T | 1:1000 |
