# Supplementary material for: Assessment of STAT5 as a potential therapy target in enzalutamide-resistant prostate cancer
Source: PLoS One. 2020 Aug 13;15(8):e0237248. doi: 10.1371/journal.pone.0237248 (PMC7425943; doi:10.1371/journal.pone.0237248)

Figure 2A STAT5a/b

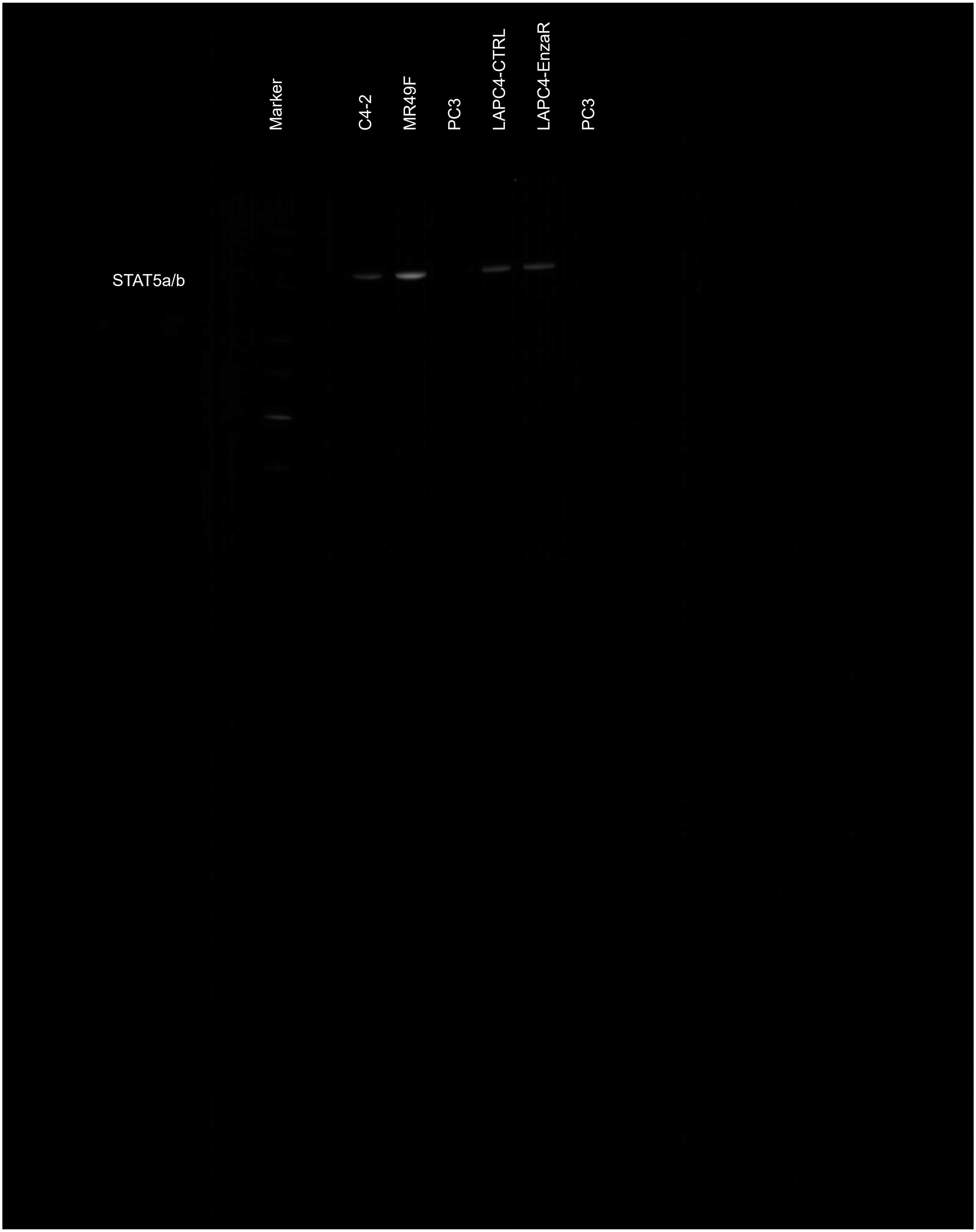

Figure 2A GAPDH

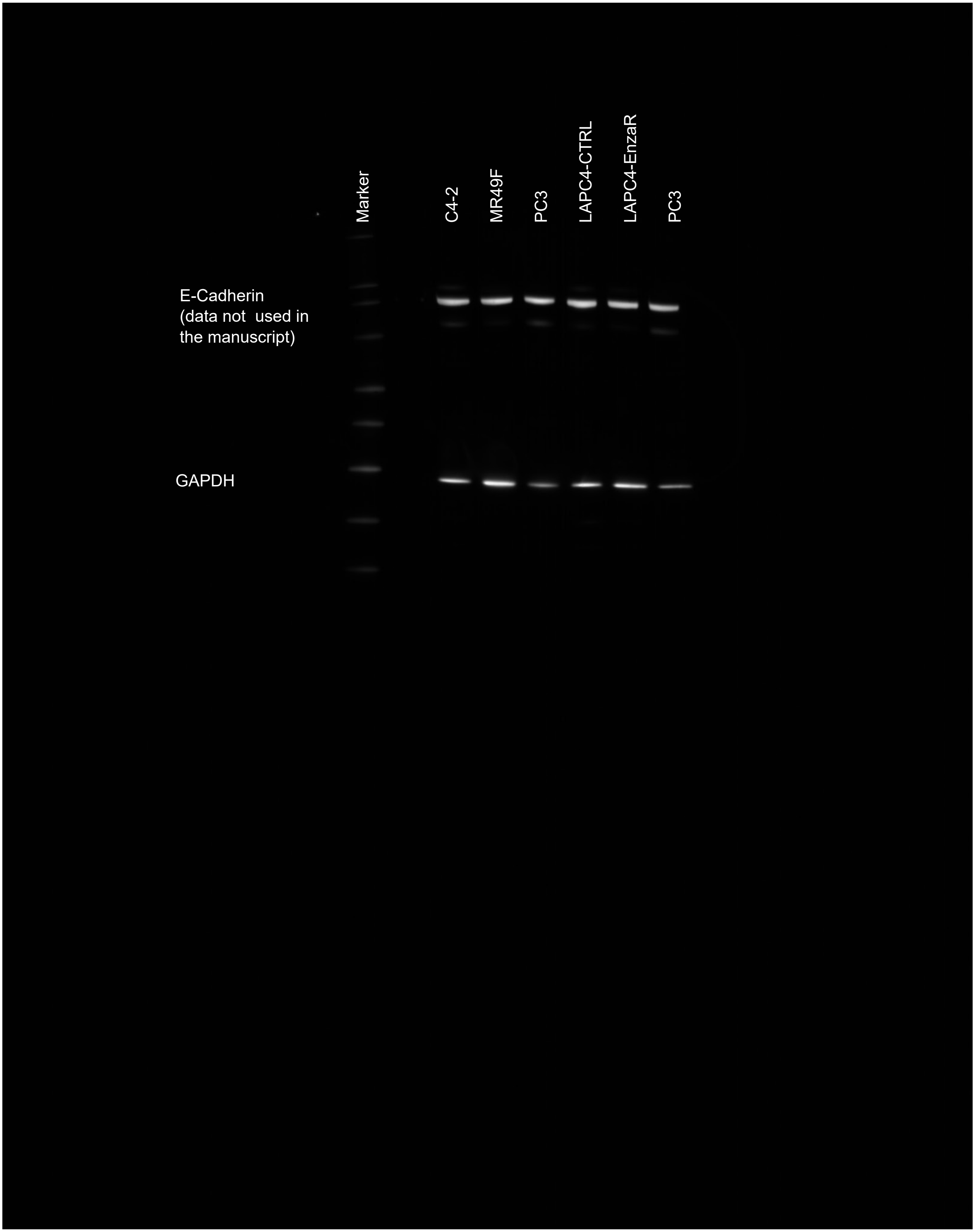

Figure 2C STAT5a/b short exposure time

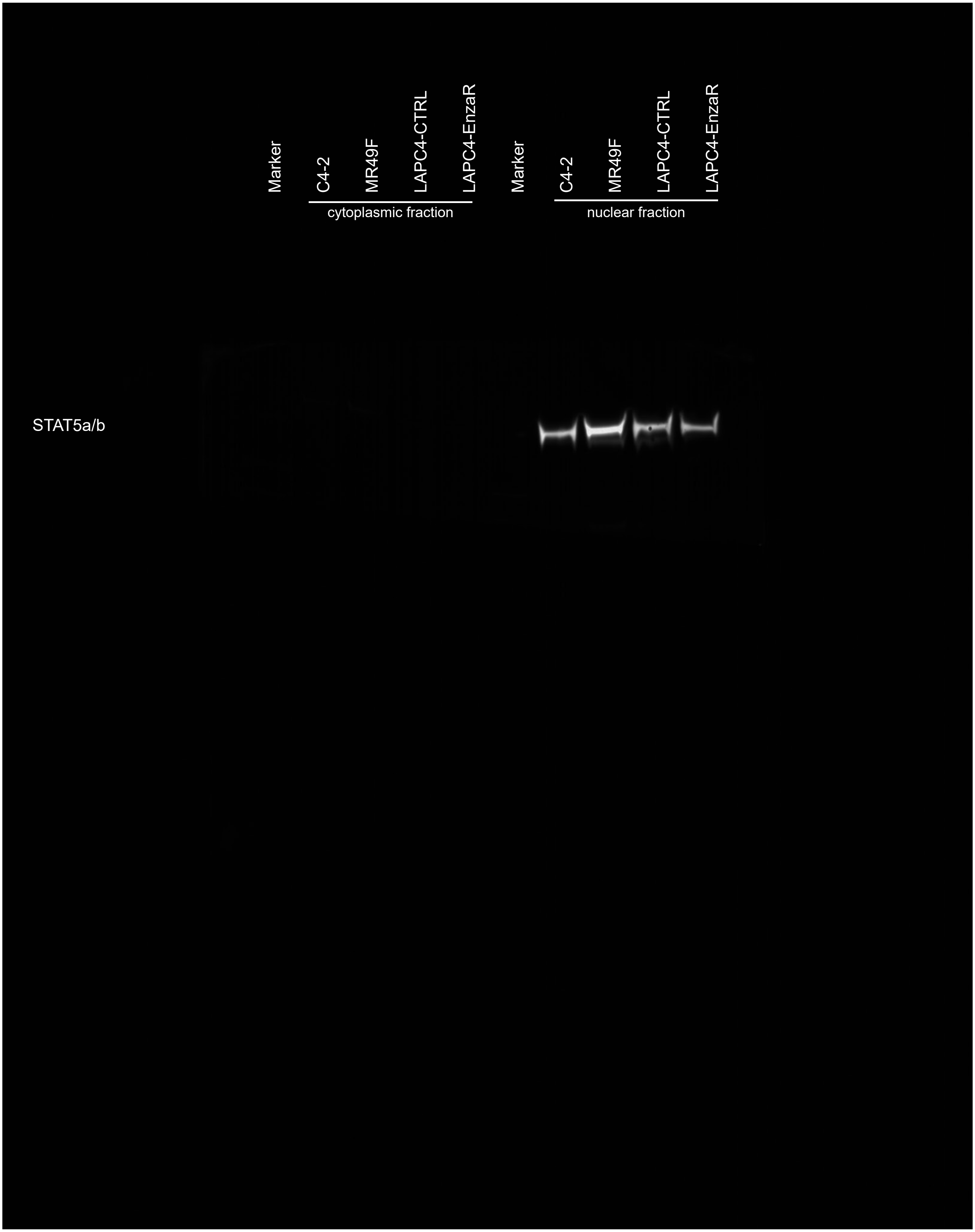

Figure 2C STAT5a/b long exposure time

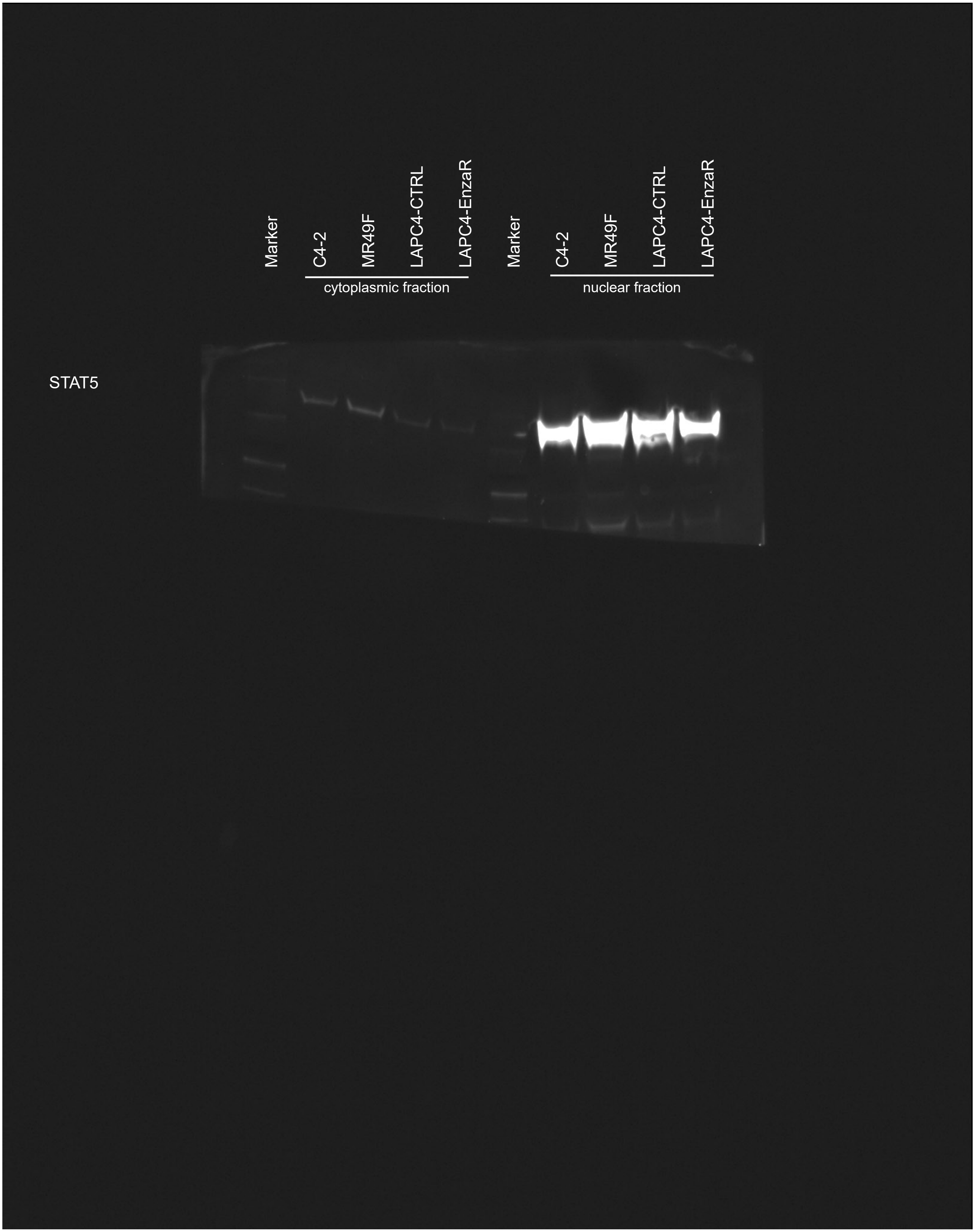

Figure 2C Lamin A/C and GAPDH

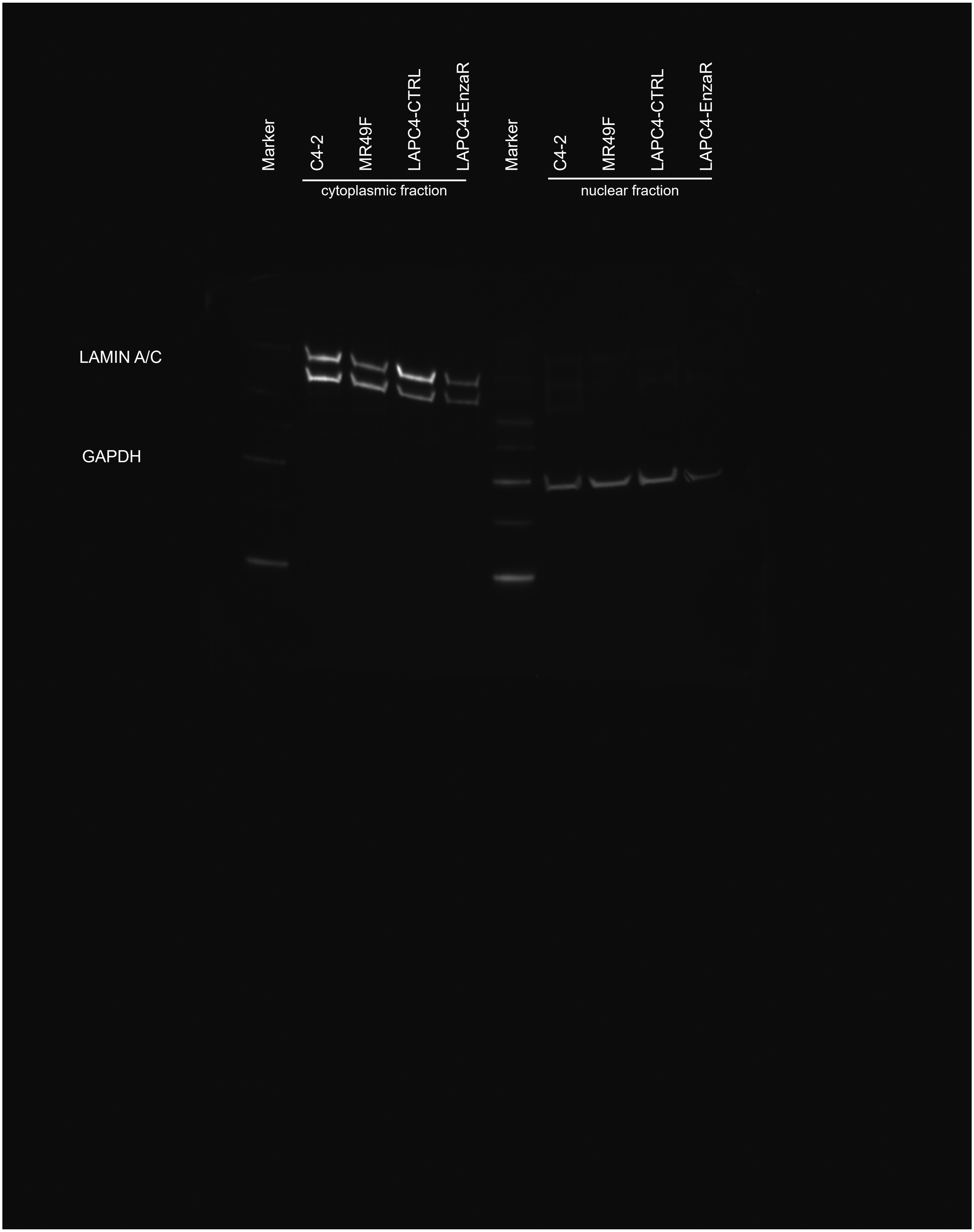

Figure 2G AR

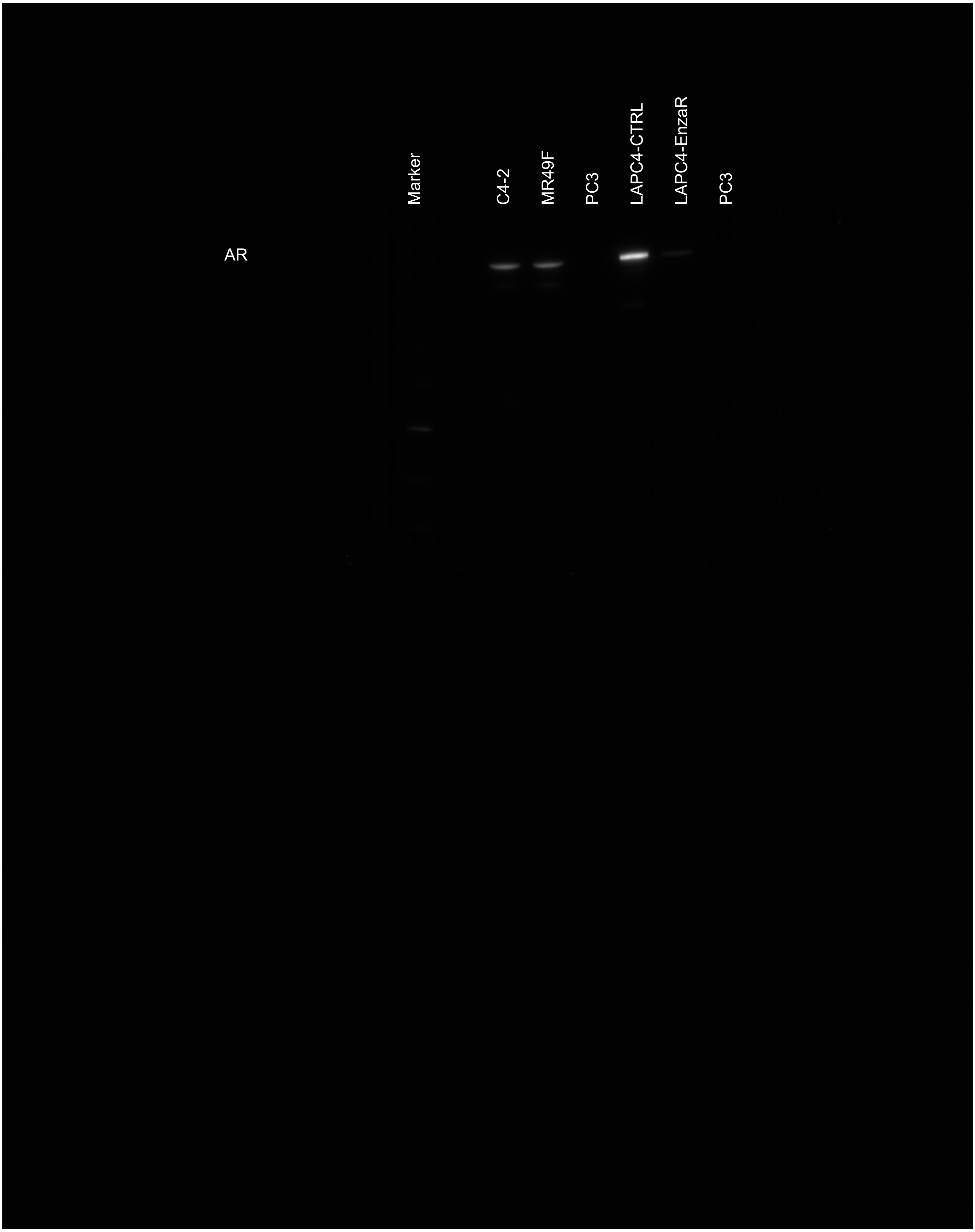

Figure 2G PSA

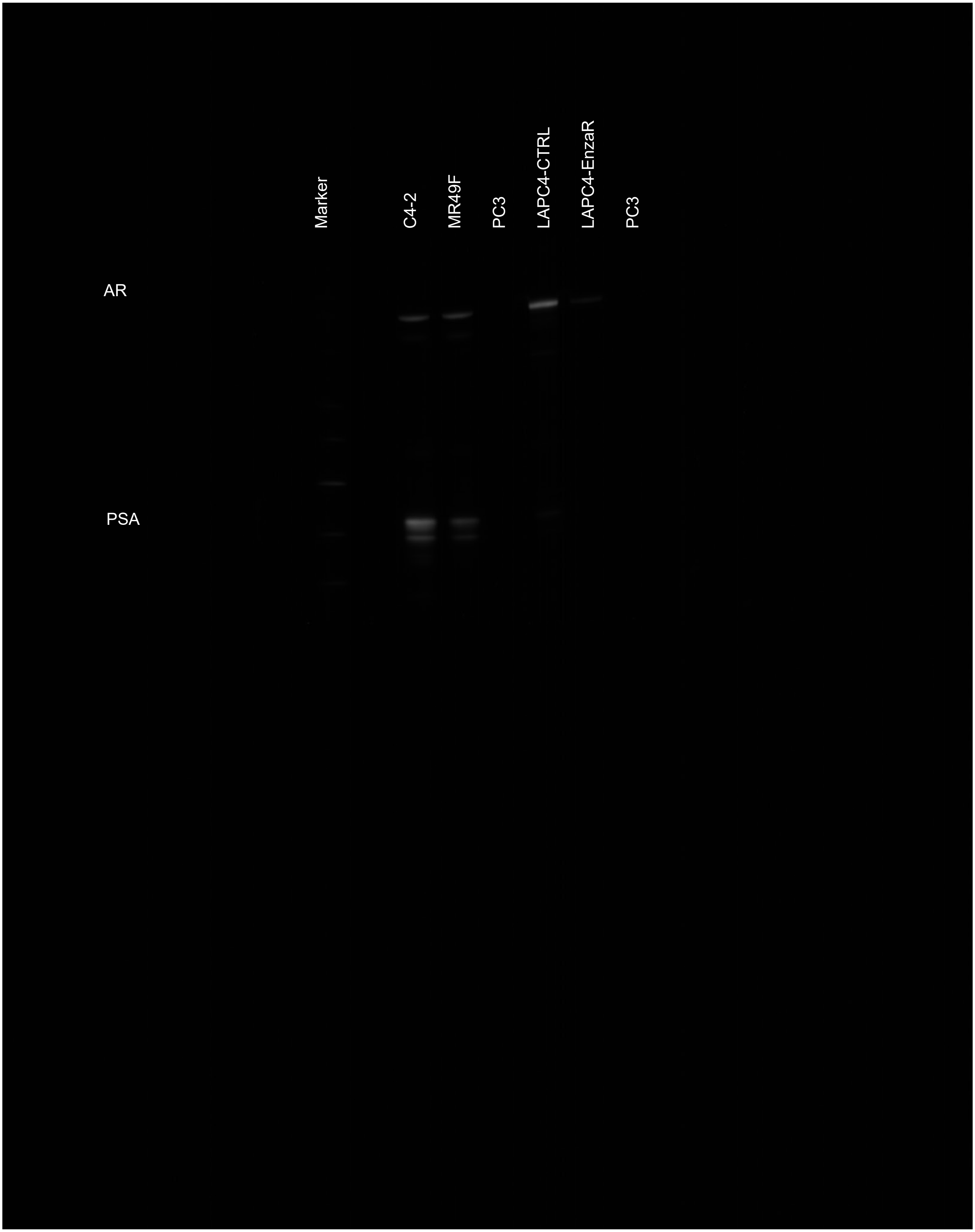

Figure 2G GAPDH

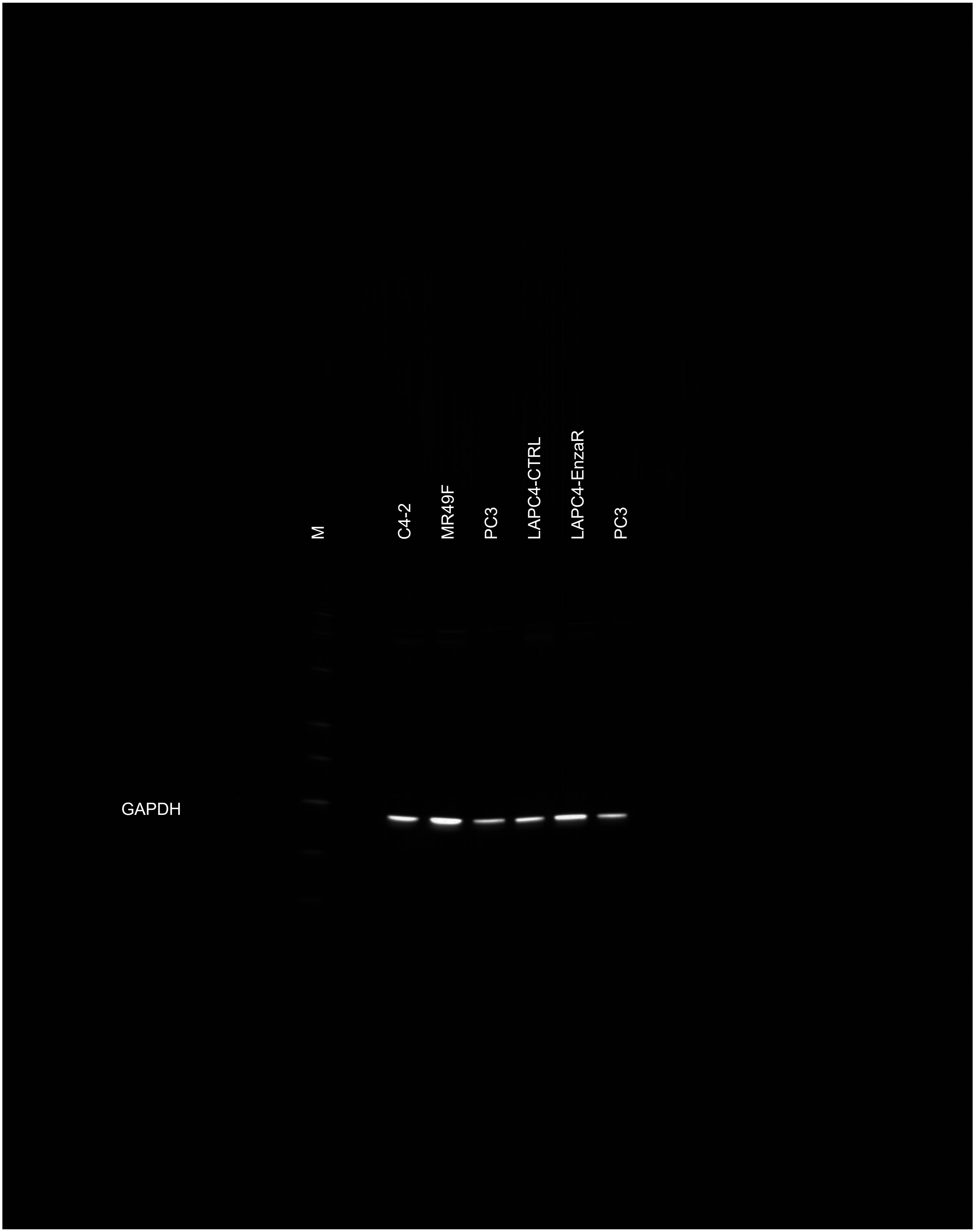

S2 Fig B: GAPDH

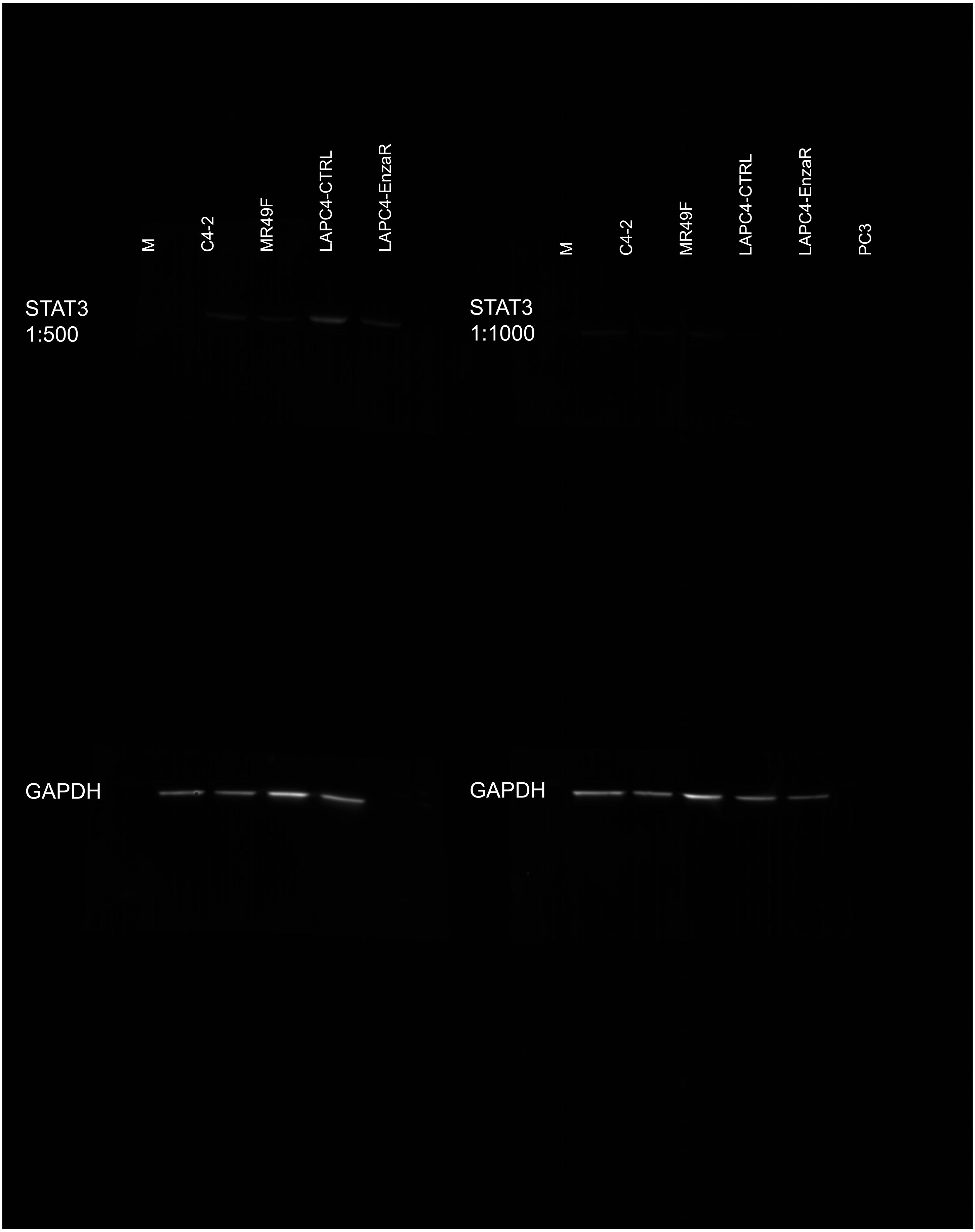

S2 Fig B: STAT3

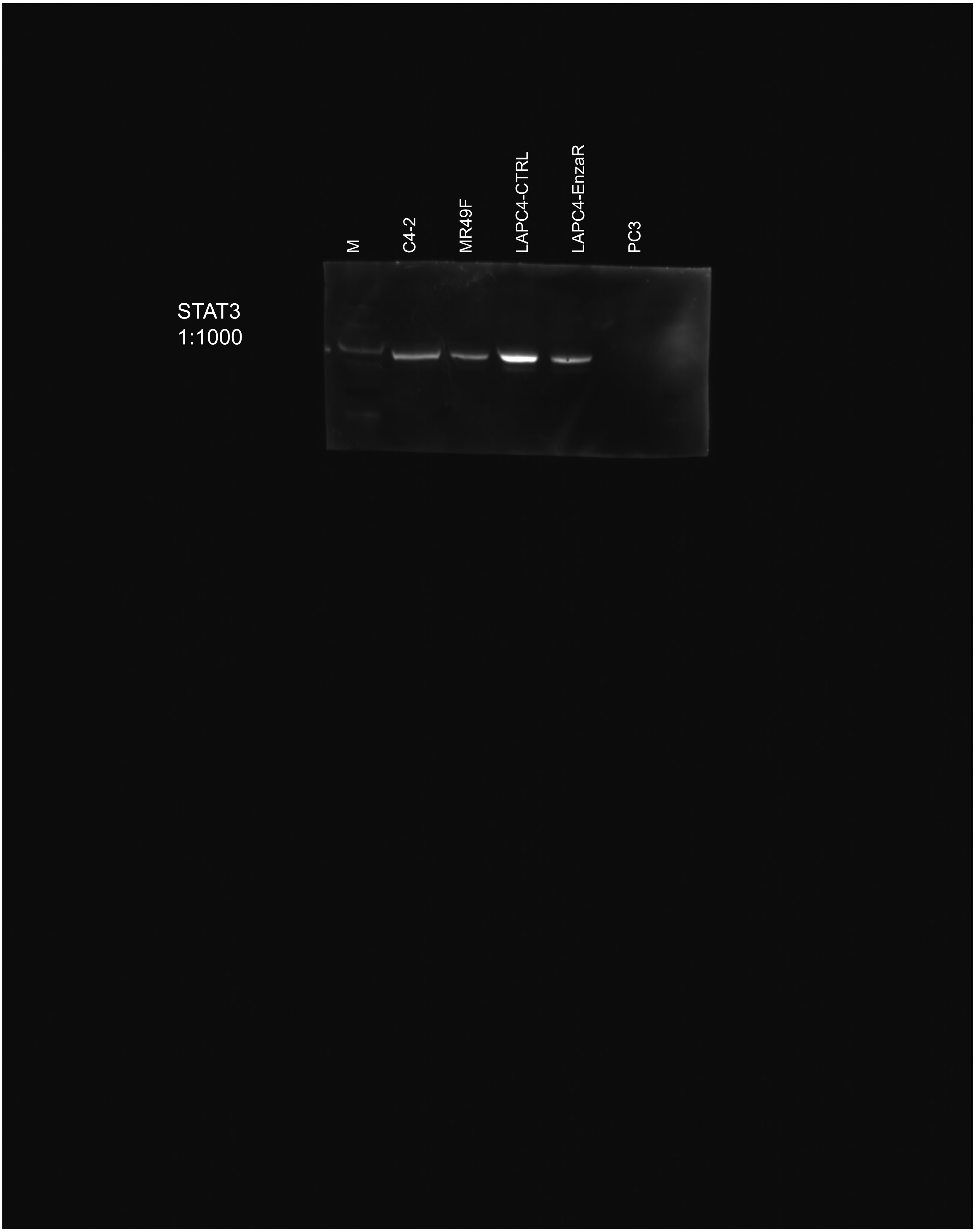

S2 Fig C: LAPC4: pSTAT3, STAT3, GAPDH
(Odyssey CT)

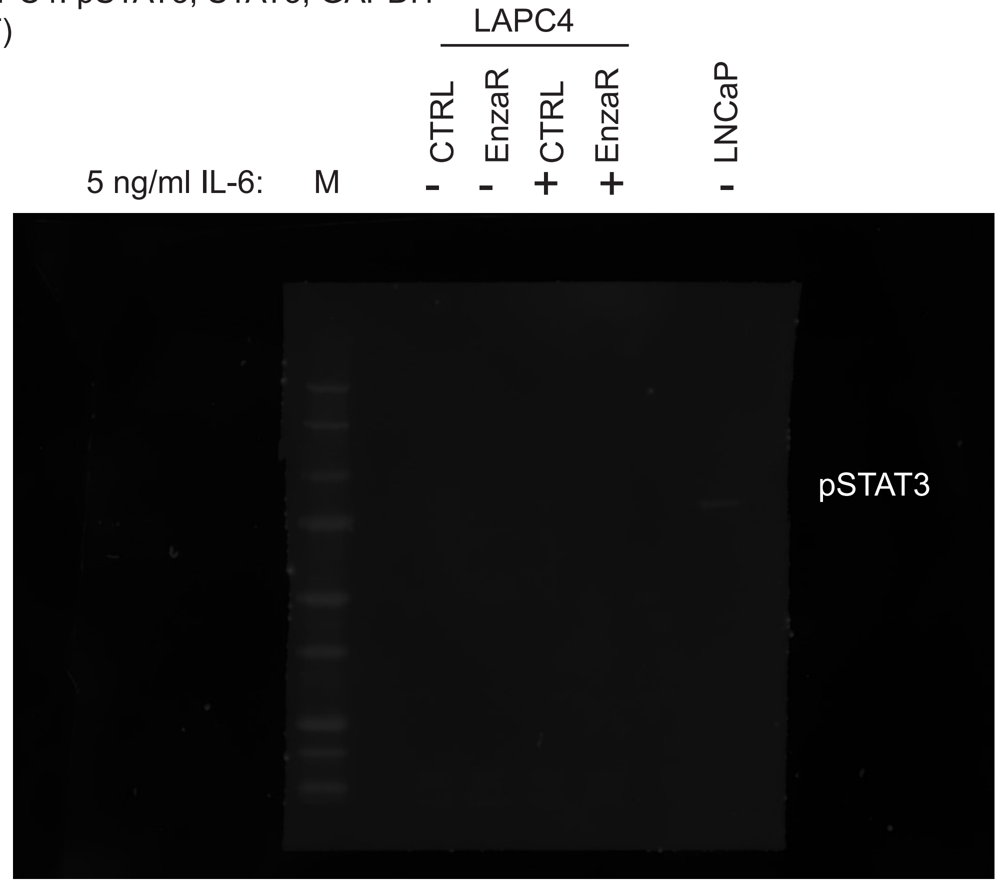

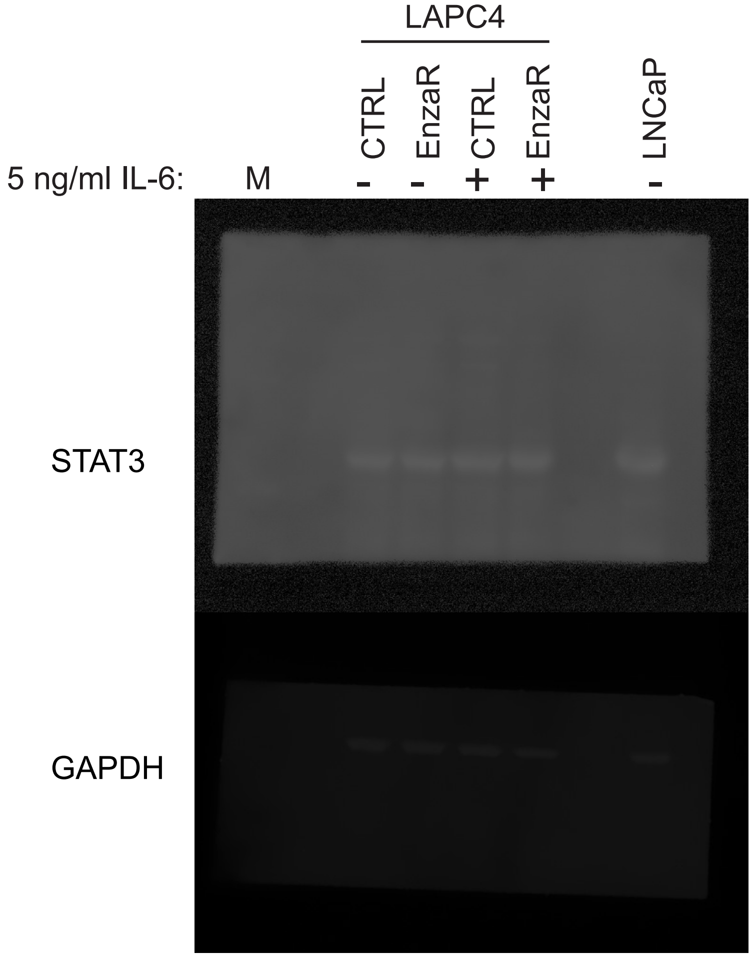

S2 Fig C: LNCaP-ABL+DuCaP: pSTAT3, STAT3, GAPDH (Odyssey CT)

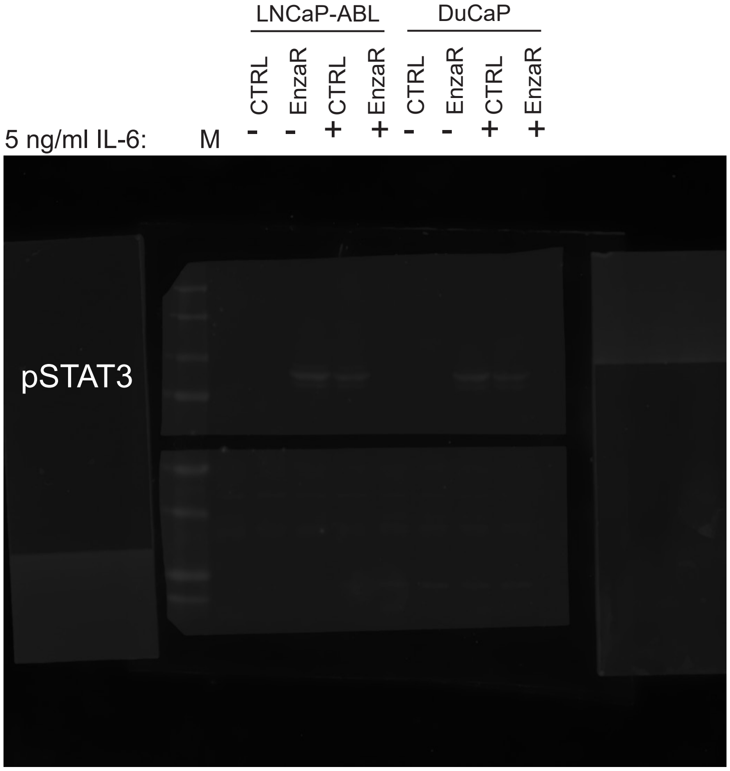

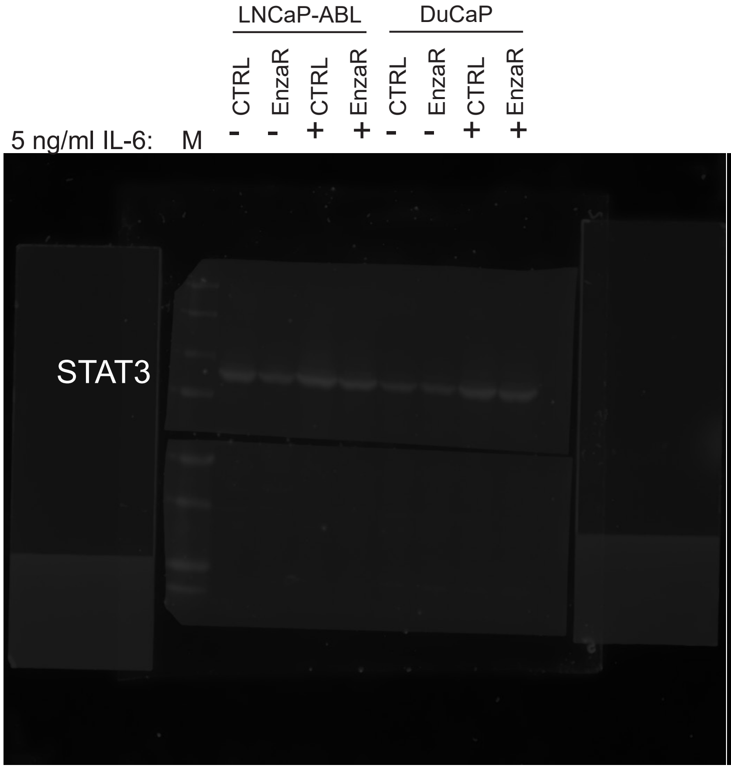

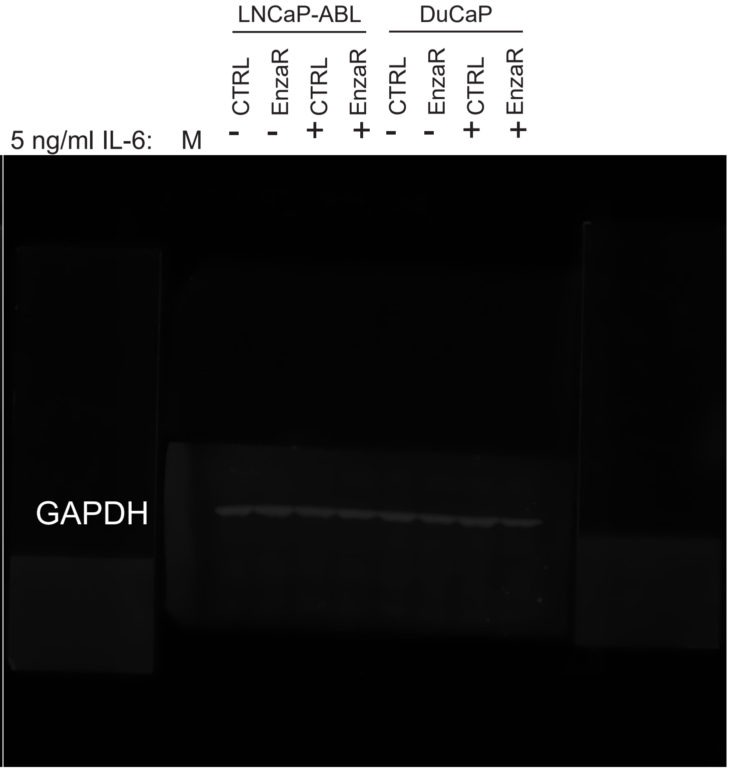

S3 Fig A: STAT5 and GAPDH

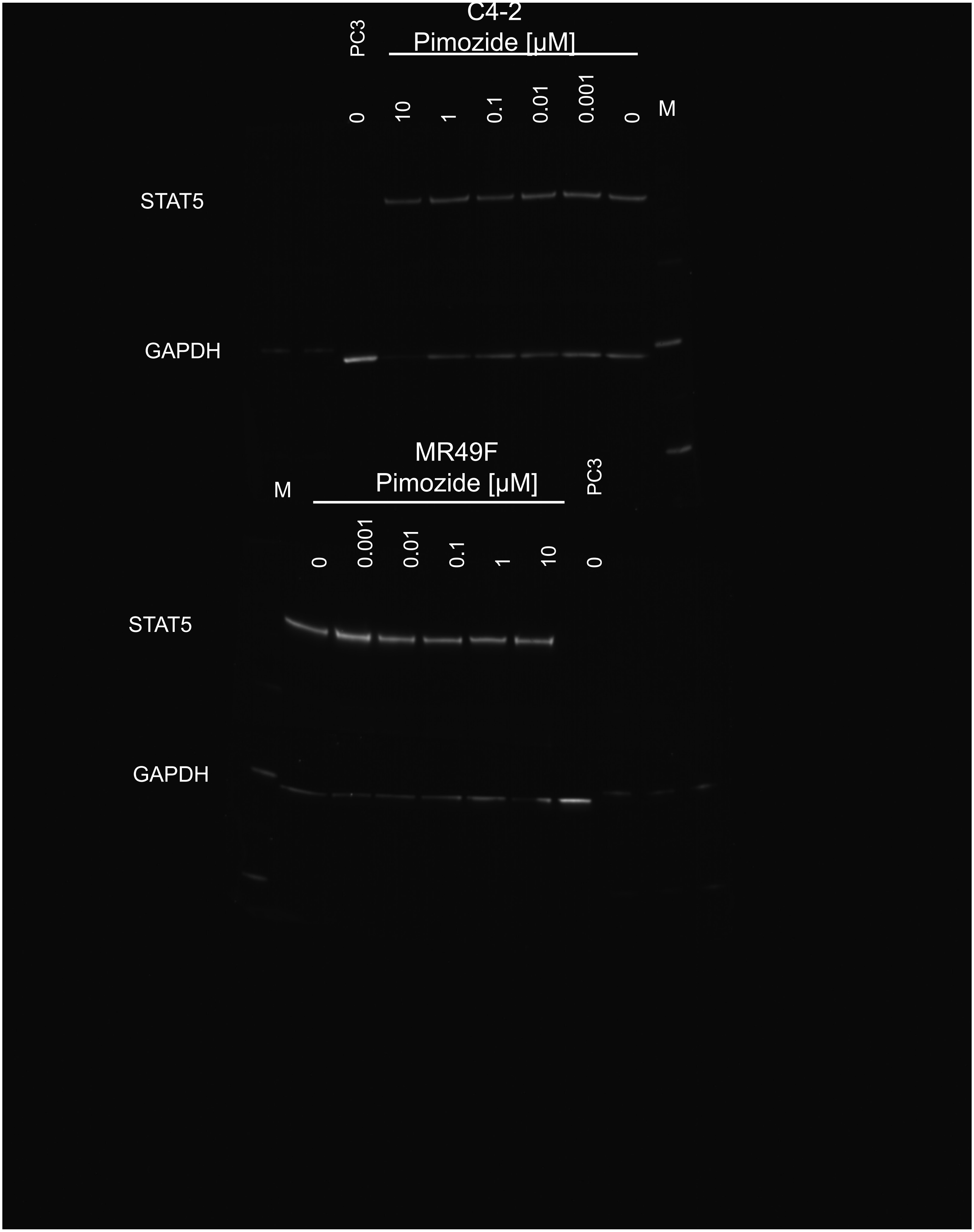

S3 Fig B + S4 Fig B: LAMIN A/C

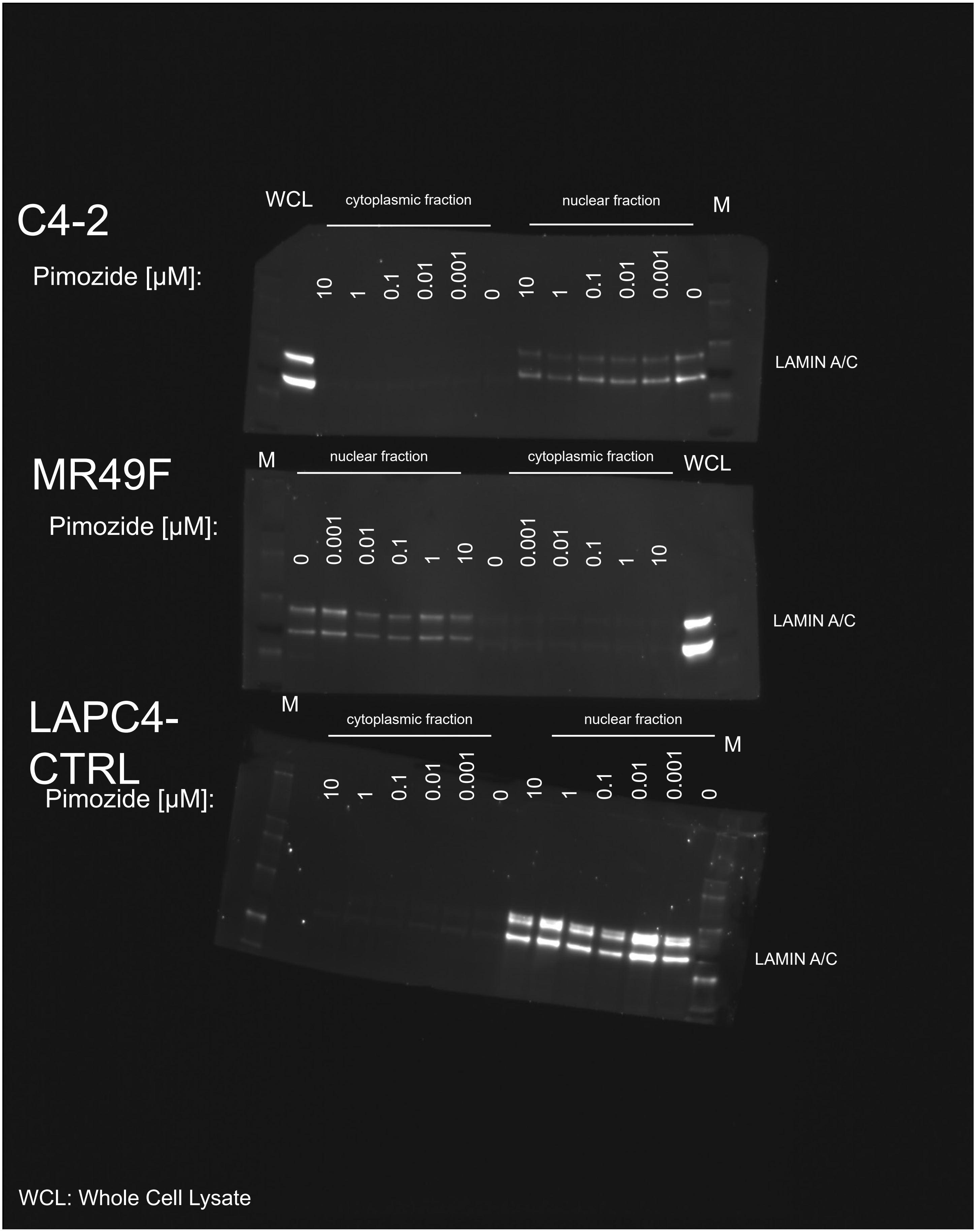

S3 Fig B: GAPDH

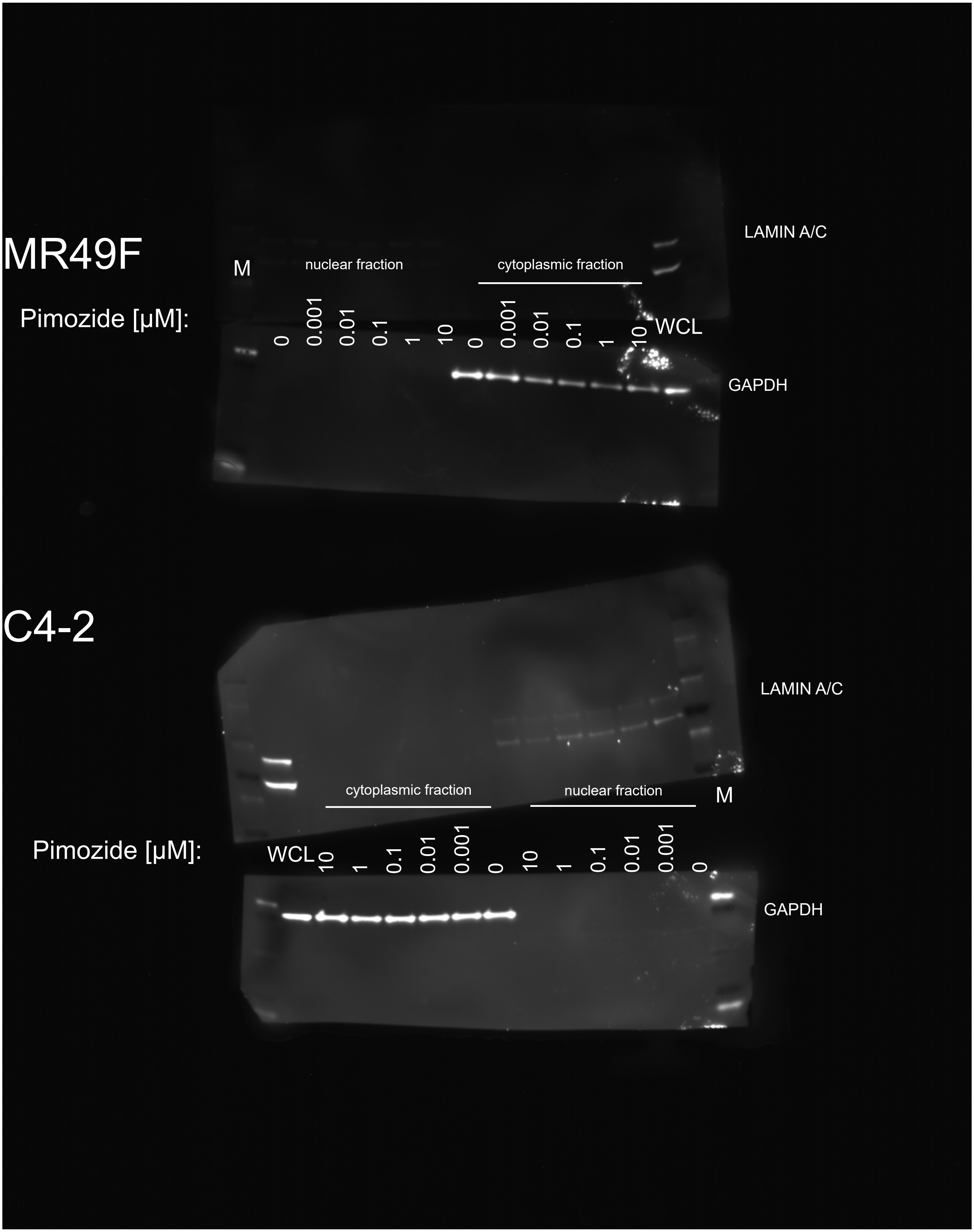

S3 Fig C: STAT5

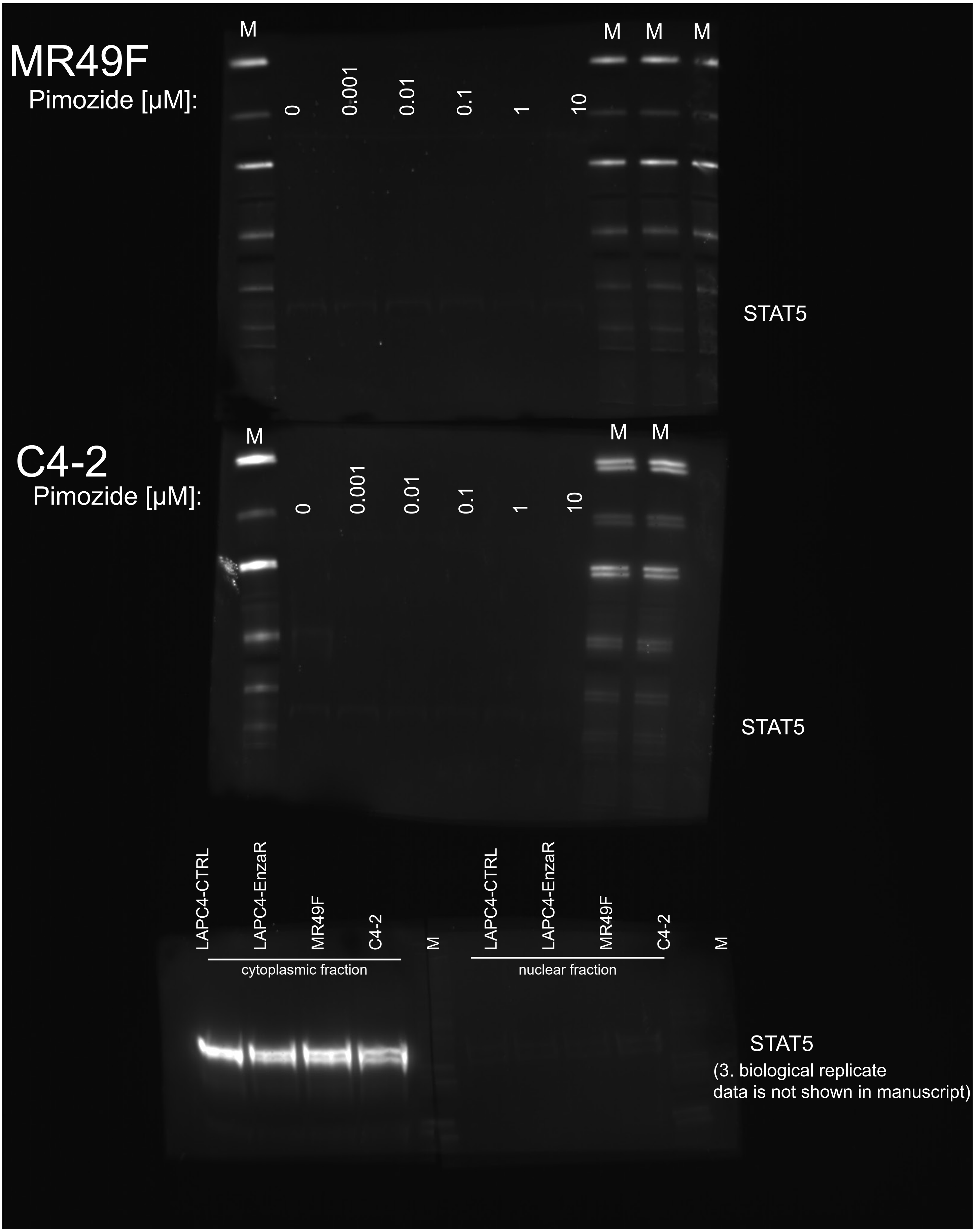

S3 Fig C: LAMIN A/C

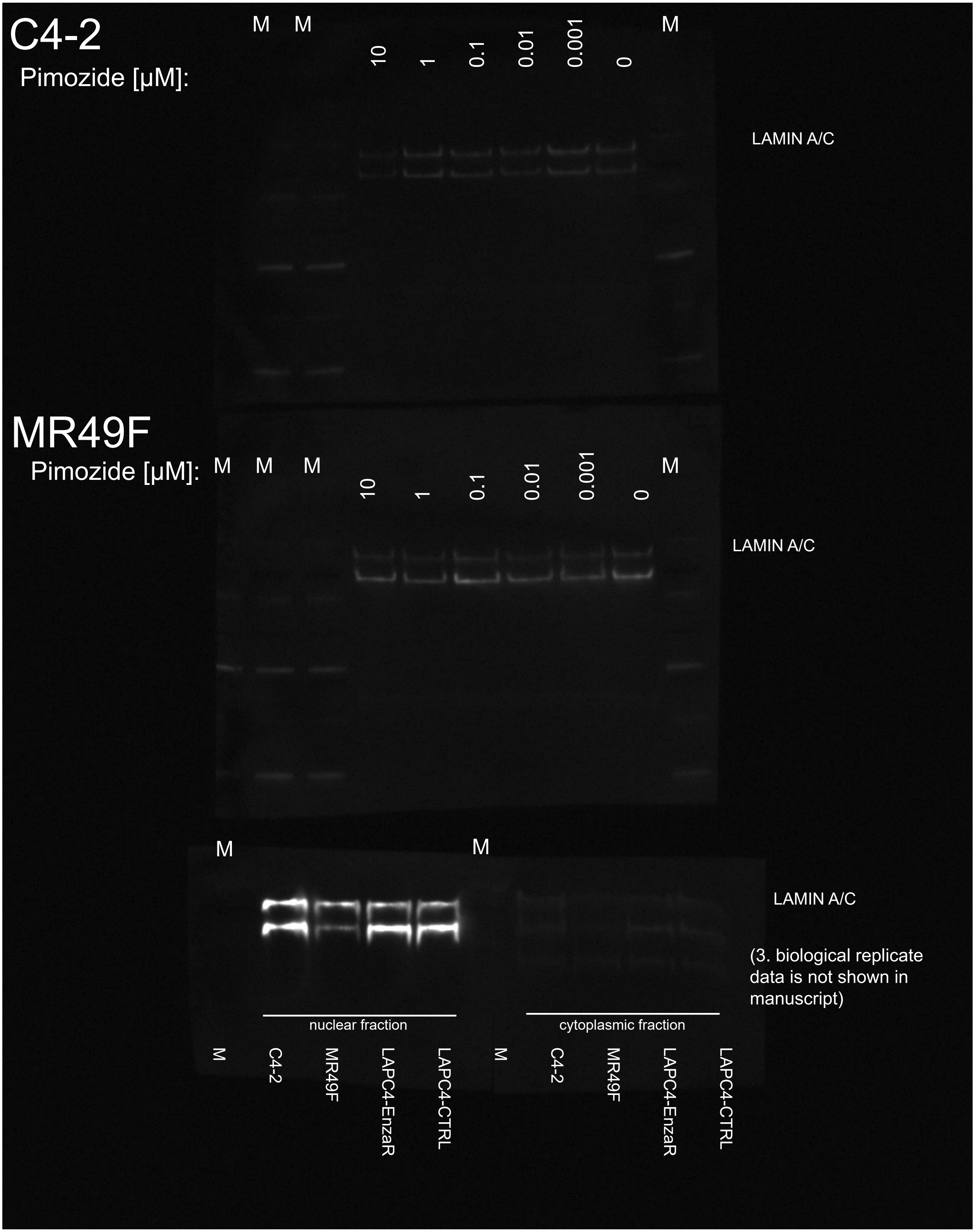

S4 Fig B+A: LAMIN A/C

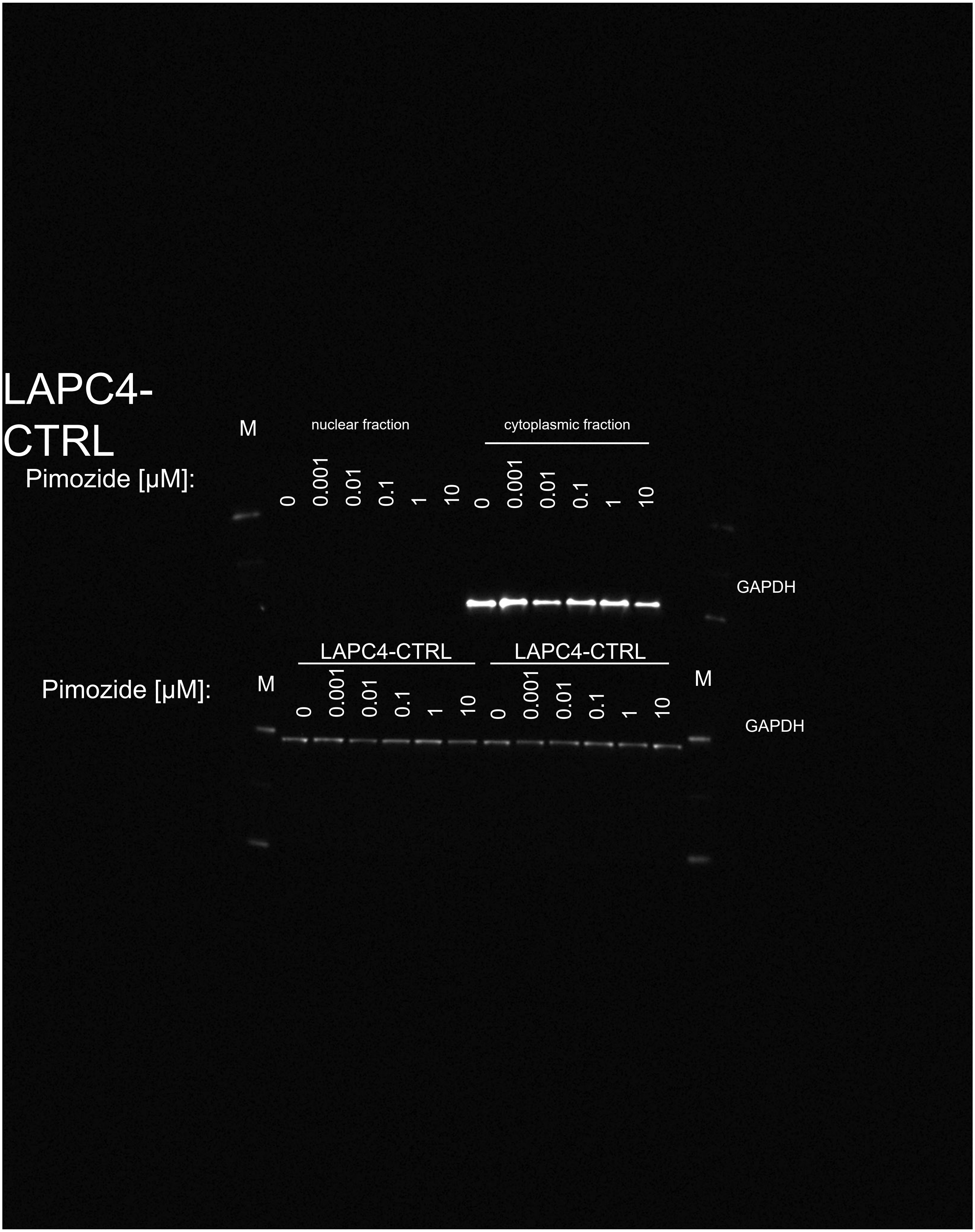

S4 Fig A: STAT5

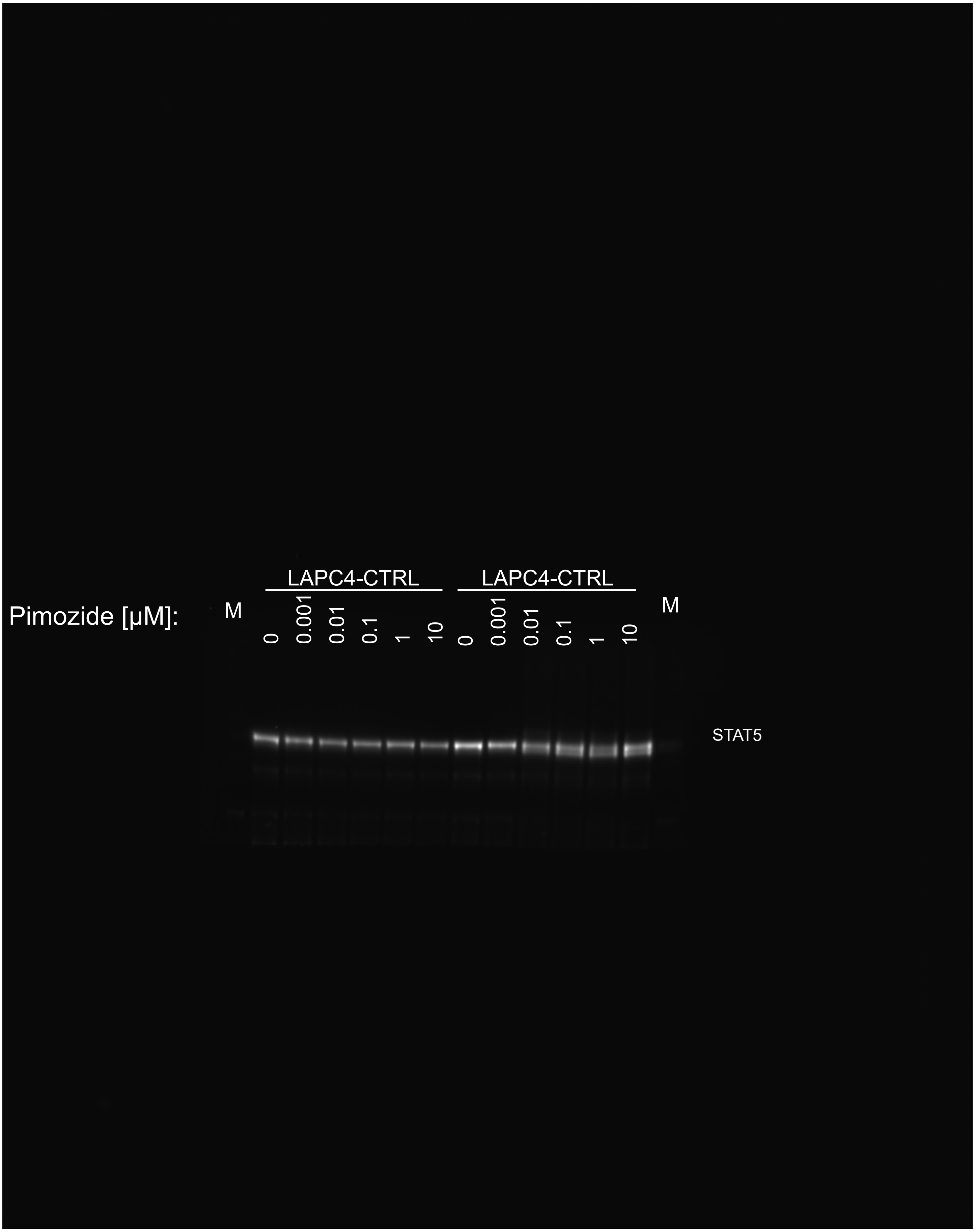

S4 Fig B: LAMIN A/C and GAPDH

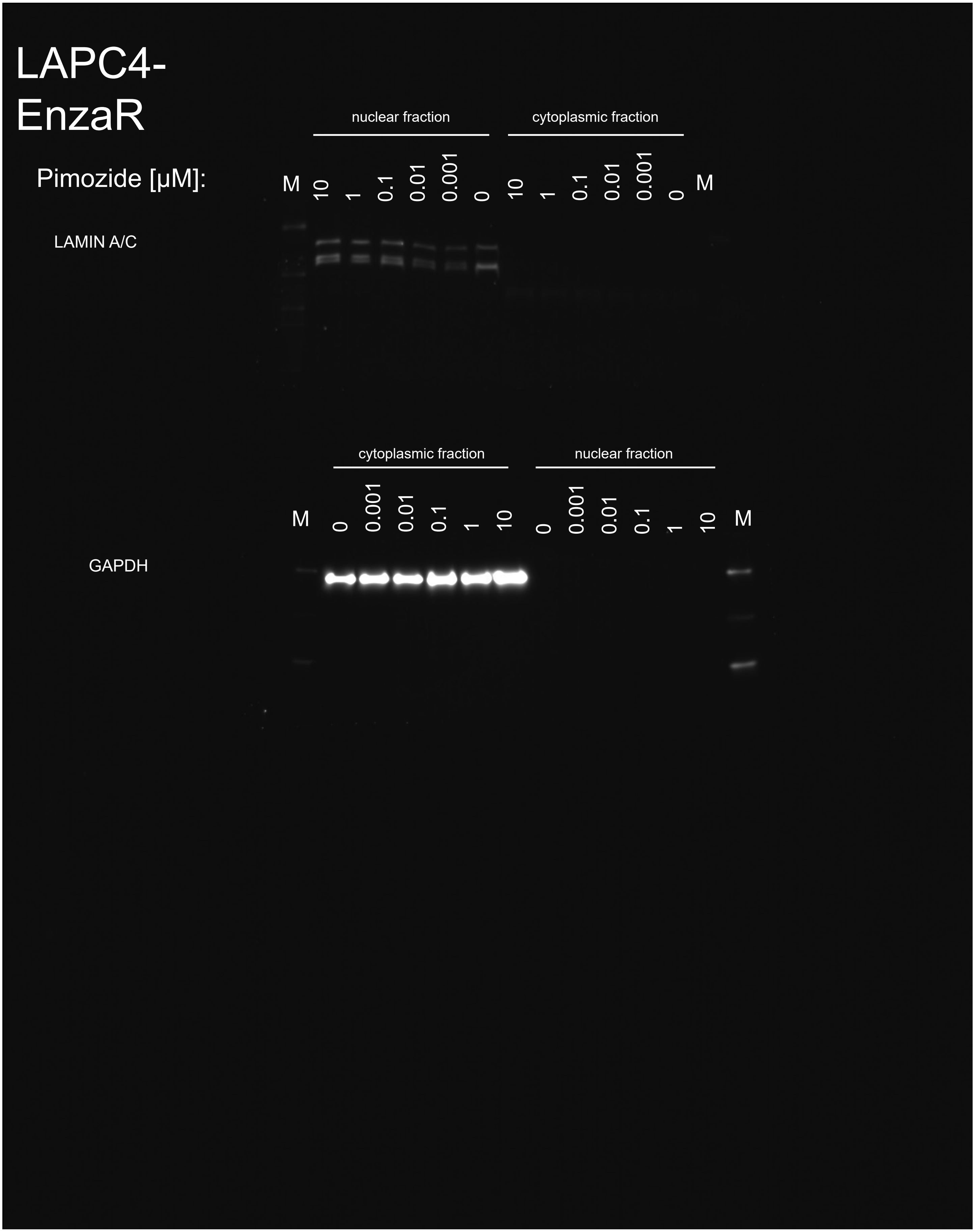

S4 Fig C: LAMIN A/C

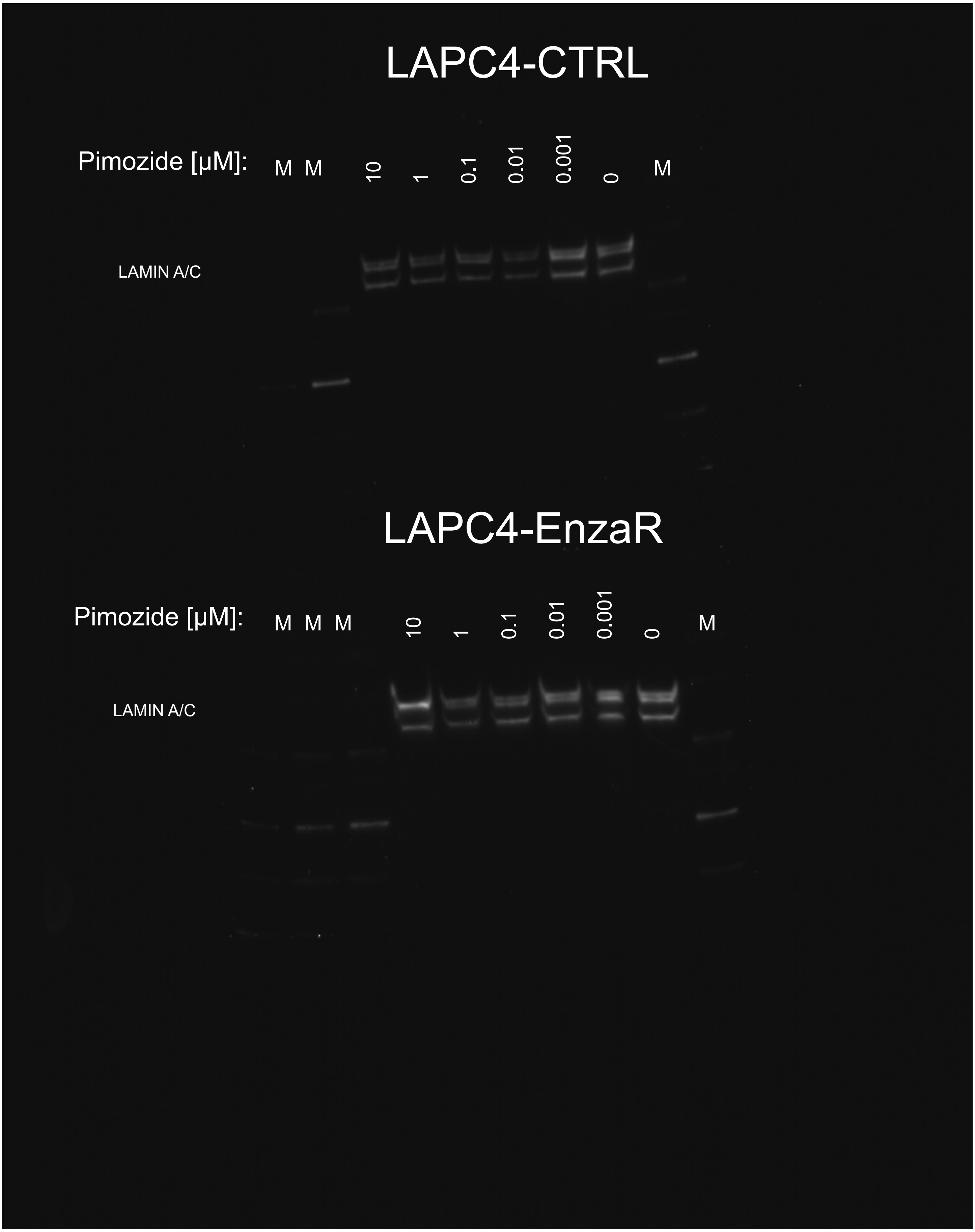

S4 Fig C: STAT5

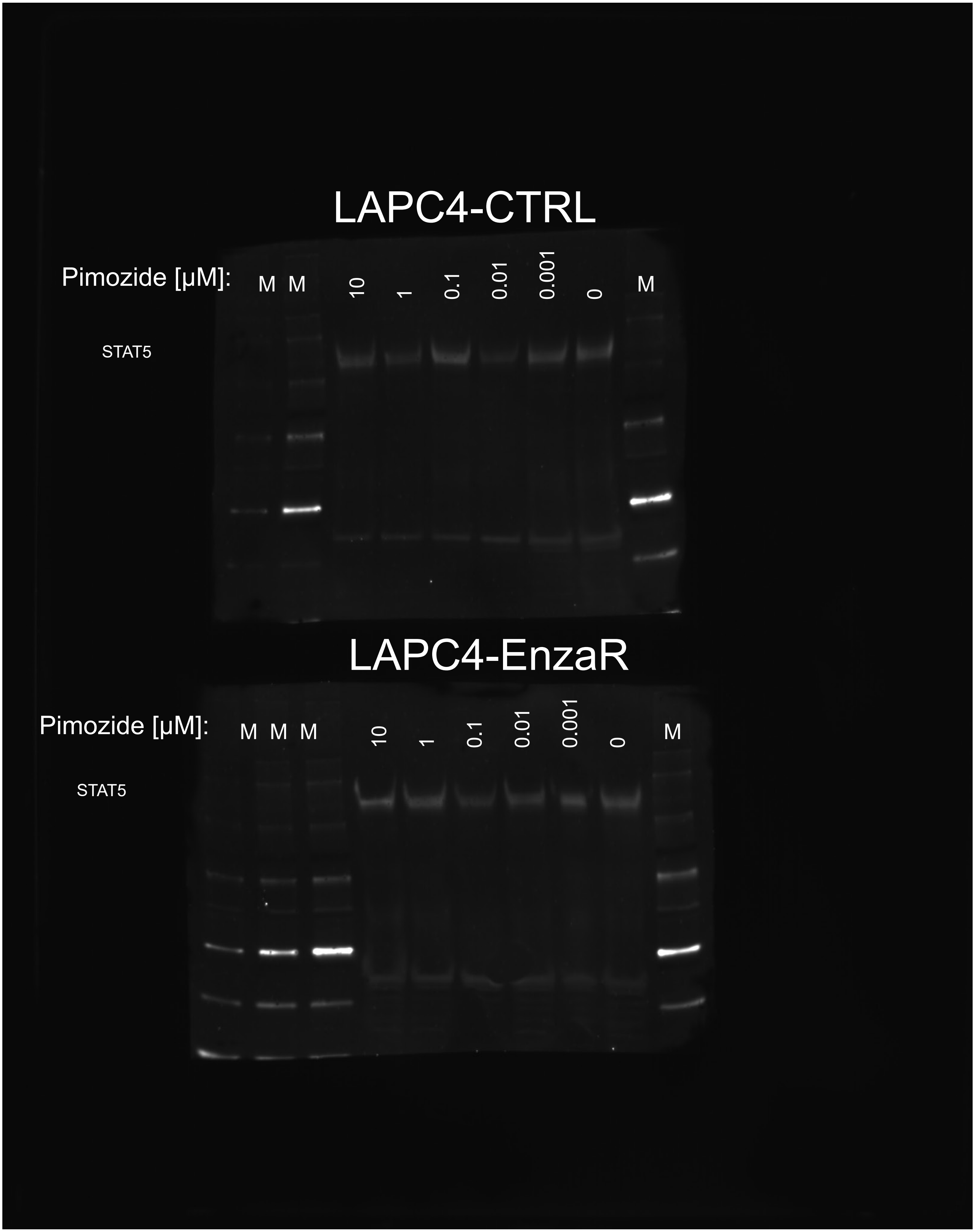

S6 Fig E: STAT5

Final siRNA concentration used per well: 25 nM 125 nM

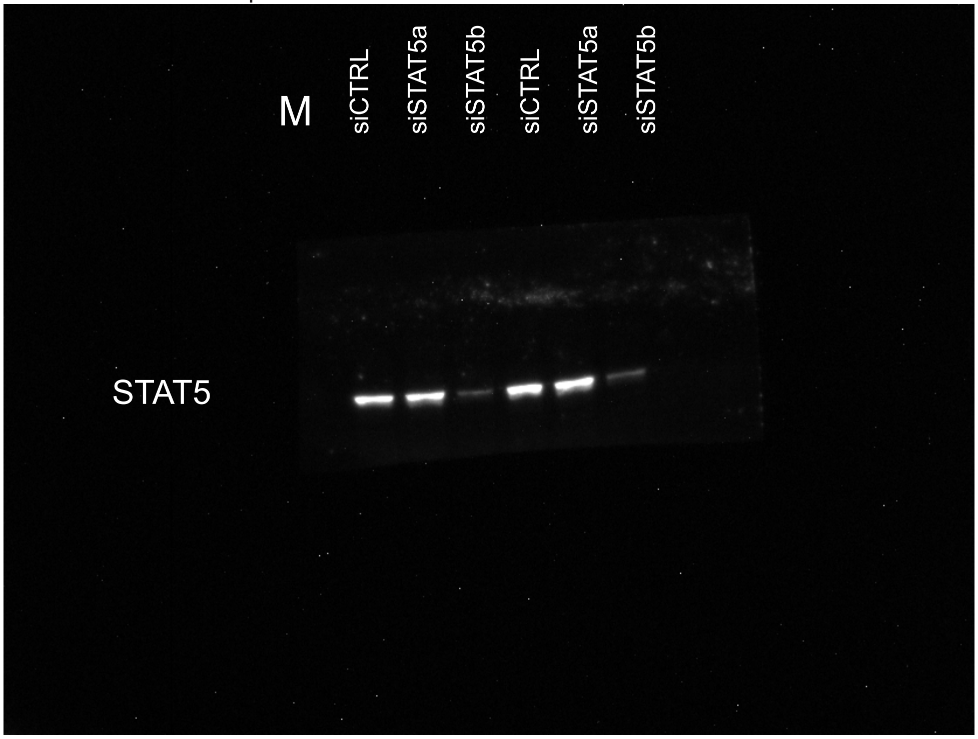

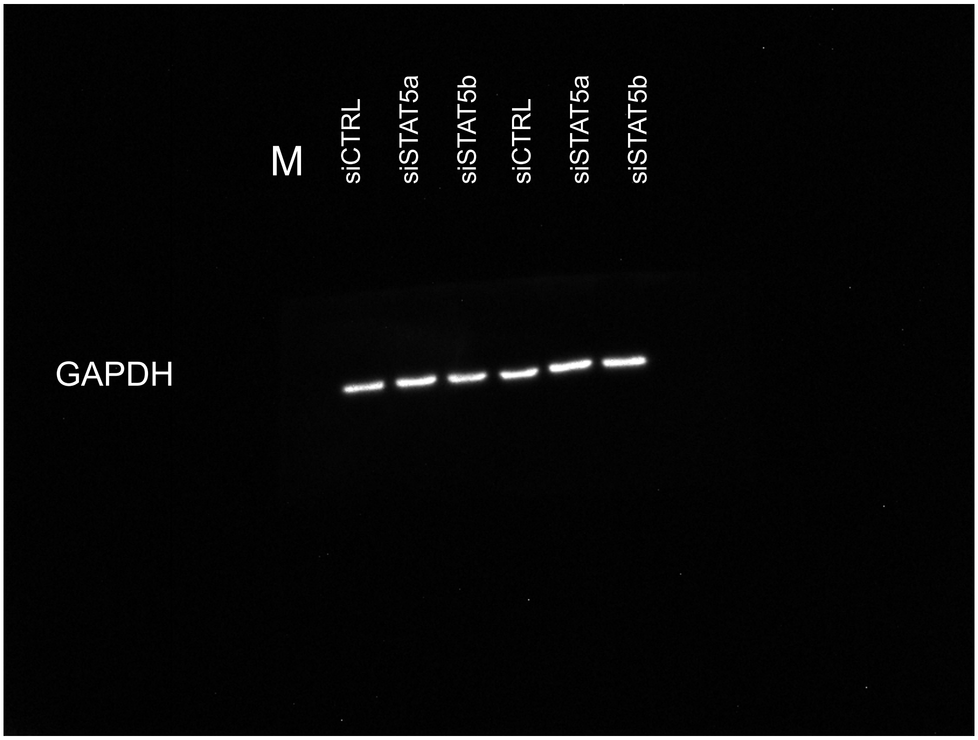

S6 Fig F: GAPDH

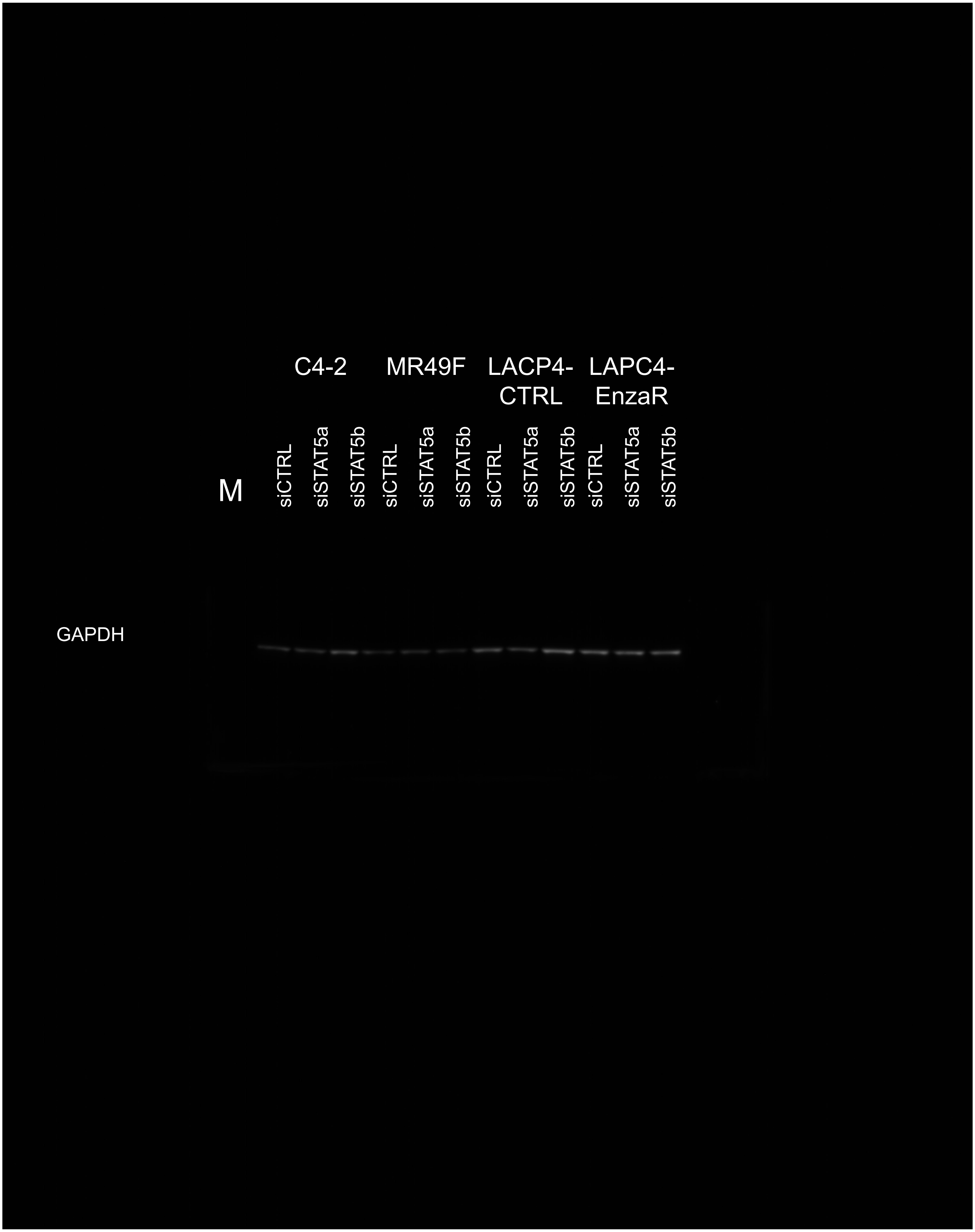

S6 Fig F: STAT5

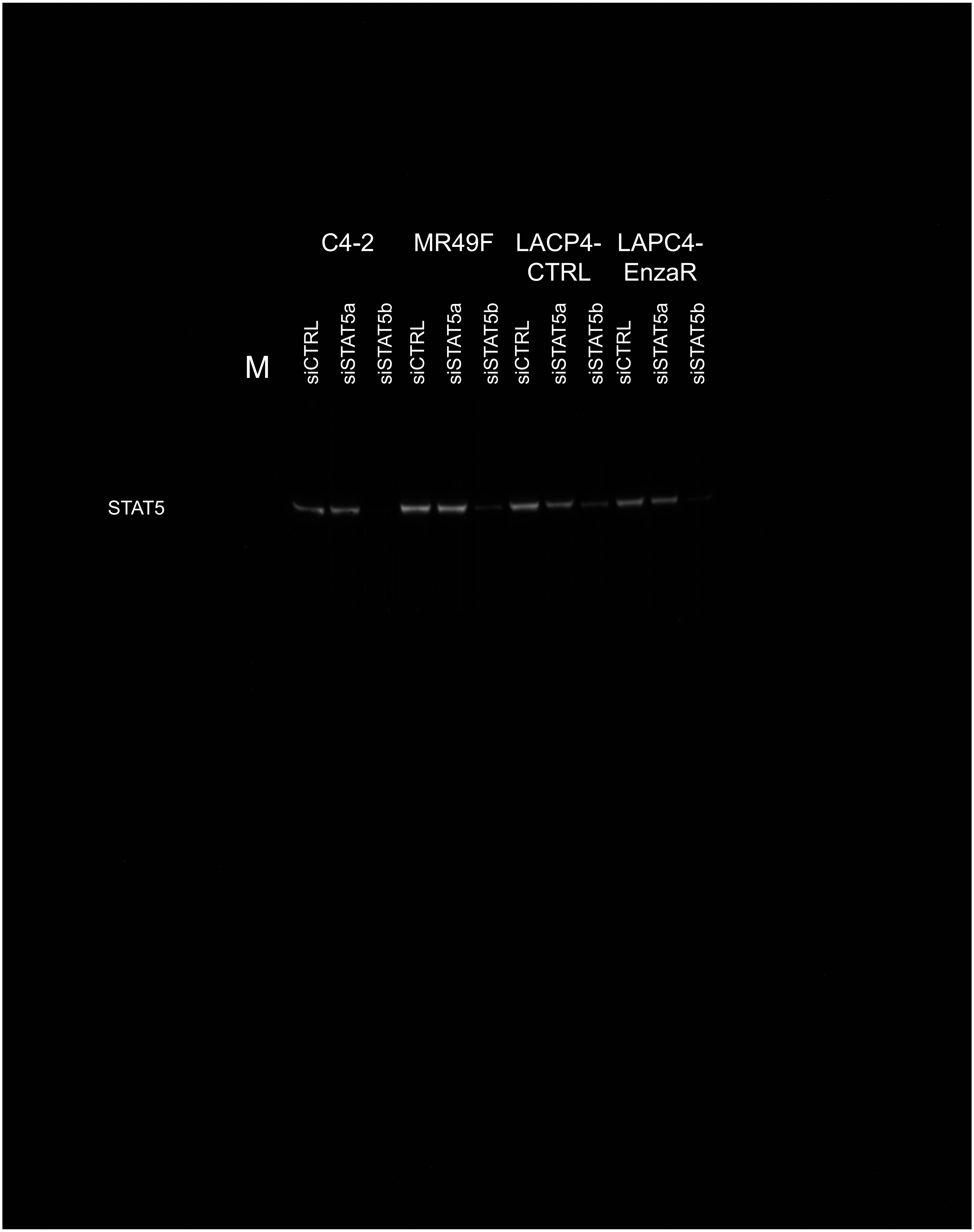

Supplement: S1 Raw images — (PDF) [file pone.0237248.s009.pdf]
